# Discovery of dual tubulin-NEDDylation inhibitors with antiproliferative activity

**DOI:** 10.1080/14756366.2022.2136173

**Published:** 2022-11-04

**Authors:** Dong-Jun Fu, Ting Wang

**Affiliations:** Beijing Research Institute of Chinese Medicine, Beijing University of Chinese Medicine, Beijing, China

**Keywords:** Tubulin, NEDDylation, molecular docking, antiproliferative activity

## Abstract

Although various dual-target tubulin inhibitors have been designed and synthesised, no dual tubulin-NEDDylation inhibitors as antiproliferative agents were reported so far. In this work, a series of trimethoxyphenyl analogues as potential dual tubulin-NEDDylation inhibitors were synthesised and evaluated for their antiproliferative activity. Among them, compound **C11** exhibited the most potent inhibitory activity with IC_50_ values of 1.17, 2.48, and 1.47 μM against HepG2, PC3, and MCF7 cells, respectively. In addition, it displayed the potent inhibitory activity against tubulin with an IC_50_ value of 2.40 μM and obviously inhibited tubulin polymerisation in HepG2 cells. Furthermore, **C11** inhibited NEDDylation by a ATP-dependent manner. Molecular docking studies revealed that the methoxy group and dithiocarbamate group of **C11** could form hydrogen bonds with residues of tubulin and E1 NEDD8-activating enzyme (NAE). These results suggested that compound **C11** was a dual tubulin-NEDDylation inhibitor with antiproliferative activity.

## Graphical Abstract

**Figure:**
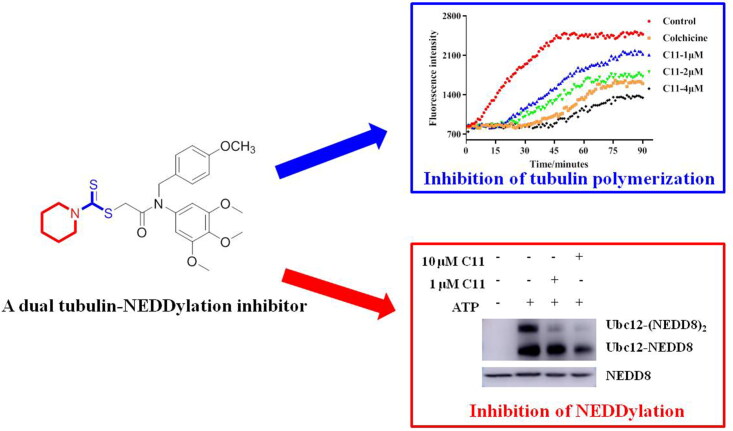

## Introduction

**Figure 1.**
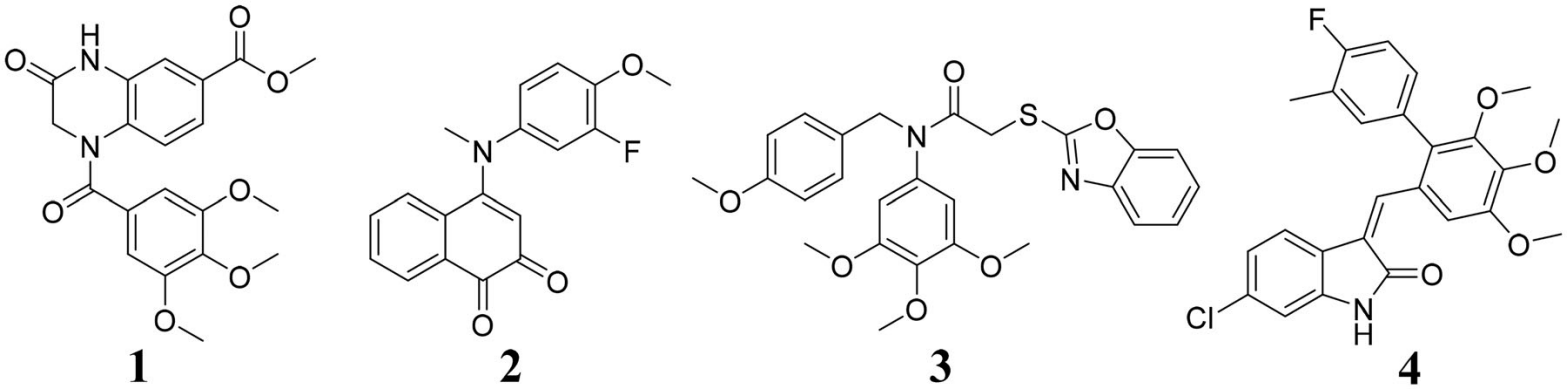
Tubulin polymerisation inhibitors as anticancer agents.

Microtubules as dynamic protein polymers of *α*- and *β*-tubulin play essential roles in a range of cellular processes, such as intracellular transport, cell division, cell growth, cell shape maintenance, and cell motility^1^. Recently, microtubule-targeting agents interacted with tubulin have been widely developed for cancer therapy^2^. *N*-substituted 3-oxo-1,2,3,4-tetrahydro-quinoxaline-6-carboxylic acid derivative **1** (Figure 1) inhibited tubulin polymerisation and exhibited the potent antiproliferative activity^3^. Tubulin polymerisation inhibitor **2** could induce intracellular reactive oxygen species (ROS) production and mitochondrial depolarization^4^. Compound **3** induced the G2/M phase arrest and cell apoptosis against MGC803 cells by targeting tubulin^5^. Tubulin polymerisation inhibitor **4** could interrupt the formation of microtubule network by inhibiting the tubulin polymerisation with an IC_50_ value of 4.96 μM^6^. Thus, tubulin has become an important target for the development of anticancer agents^7^.

**Figure 2.**
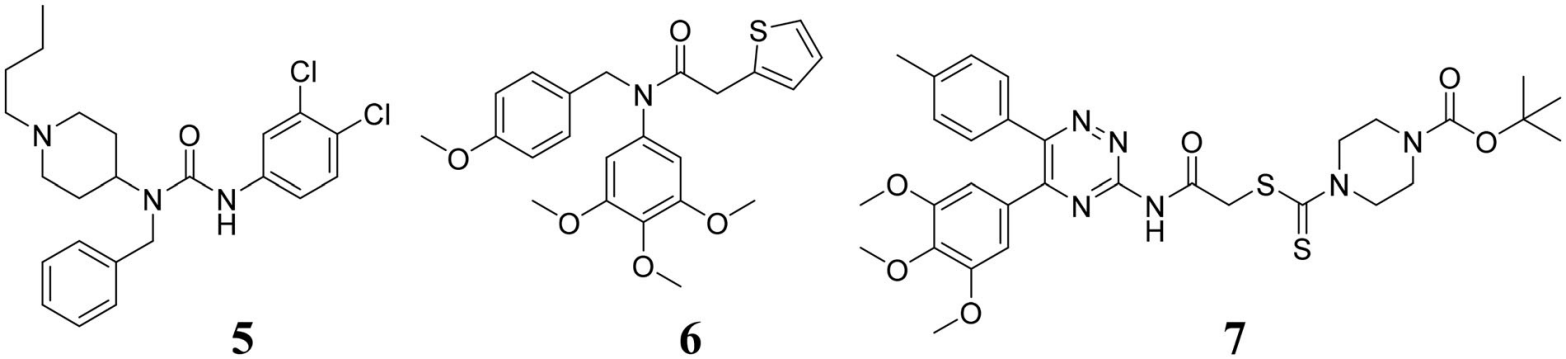
Representative NEDDylation modulators.

NEDDylation is catalysed by three-enzyme cascade including the E1 NEDD8-activating enzyme (NAE), E2 NEDD8-conjugating enzyme and E3 NEDD8 ligase, which could lead to attachment of ubiquitin-like NEDD8 to a substrate protein on a lysine residue^8^. Many studies have illustrated the close relationship between NEDDylation in multiple pathophysiological processes and different NEDDylation modulators were designed^9^. Compound **5** (Figure 2) as an orally bioavailable analogue inhibited both DCN1-mediated cullin neddylation and the DCN1-UBE2M protein-protein interaction^10^. Our group reported a novel tertiary amide derivative **6** as the NEDDylation activator to inhibit tumour progression *in vitro* and *in vivo*^11^. Compound **7** targeting NEDDylation displayed the potent activity MGC-803, EC-109, and PC-3 cells with IC_50_ values of 2.35, 10.1, and 5.71 μM^12^. All these findings showed that NEDDylation modulators might be potential anticancer agents.

**Figure 3.**
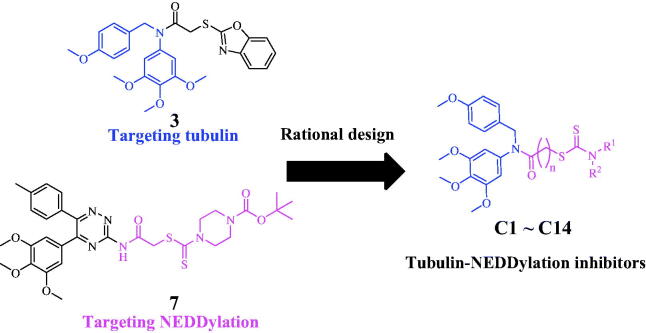
Rational design of tubulin-NEDDylation inhibitors.

In recent years, developing dual-target inhibitors has been a new trend in tumour treatment^13^. Dual-target tubulin inhibitors are novel chemical entities that could bind to tubulin and the other different target in medicinal chemistry^14^. However, there are none tubulin-NEDDylation inhibitors reported as antiproliferative agents. In this work, a series of dual tubulin-NEDDylation inhibitors were designed, synthesised and evaluated for their antiproliferative activities against HepG2, PC3, and MCF7 cell lines. As shown in Figure 3, a molecular hybridisation strategy based on the structures of tubulin polymerisation inhibitor 3 and NEDDylation modulator 7 to produce the potential tubulin-NEDDylation inhibitors. Importantly, compound **C11** was identified as a dual tubulin-NEDDylation inhibitor with potent antiproliferative activities.

## Materials and methods

### Chemistry

#### General procedure for preparation of compounds B1–B3

A solution of 3,4,5-trimethoxyaniline (3 mmol), triethylamine (4 mmol), and 1-(chloromethyl)-4-methoxybenzene (3 mmol) in CH_2_Cl_2_ (12 mL) was added. After the system was stirred at room temperature for 12 h, acyl chlorides were added to form the acylation reaction. Then, it was washed with aqueous Na_2_CO_3_ and brine. Finally, crude intermediates **B1**–**B3** were prepared without the purification. The NMR datum of compounds A1 and B1–B3 were reported by our reported reference^5^.

#### General procedure for preparation of compounds C1–C15

A solution of crude intermediates **B1–B3** (1.5 mmol), CS_2_ (3 mmol), secondary amines (1 mmol), and KOH (1 mmol) in acetone (5 mL) was added. After the system was refluxed for 7 h, it was washed with aqueous Na_2_CO_3_ and brine. Then, organic system was concentrated and purified by column chromatography with *n*-hexane/ethyl acetate (8:1) to afford the compounds **C1**–**C15**.

#### 2-((4-Methoxybenzyl)(3,4,5-trimethoxyphenyl)amino)-2-oxoethyl-4-acetylpiperazine-1-carbodithioate (C1)

White powder, yield, 48.5%, m.p:56–58 °C. ^1^H NMR (400 MHz, CDCl_3_) δ 7.10 (d, *J* = 8.6 Hz, 2H), 6.74 (d, *J* = 8.6 Hz, 2H), 6.25 (s, 2H), 4.74 (s, 2H), 4.14 (s, 4H), 3.95 (s, 2H), 3.78 (s, 3H), 3.71 (s, 3H), 3.70 (m, 2H), 3.67 (s, 6H), 3.60 − 3.48 (m, 2H), 2.07 (s, 3H). ^13^C NMR (100 MHz, CDCl_3_) δ 168.33, 165.46, 158.08, 152.55, 136.96, 135.77, 129.60, 128.43, 112.66, 104.94, 59.95, 55.21, 54.26, 52.11, 44.15, 40.03, 29.92, 20.34. HRMS calculated for C_26_H_34_N_3_O_6_S_2_, [M + H]^+^ *m/z*: 548.1889, found: 548.1897.

#### Tert-butyl-4-(((2-((4-methoxybenzyl)(3,4,5-trimethoxyphenyl)amino)-2-oxoethyl)thio)carbonothioyl)piperazine-1-carboxylate (C2)

White powder, yield, 73.2%, m.p:55–57 °C. ^1^H NMR (400 MHz, CDCl_3_) δ 7.10 (d, *J* = 8.6 Hz, 2H), 6.74 (d, *J* = 8.6 Hz, 2H), 6.25 (s, 2H), 4.74 (s, 2H), 4.17 (s, 2H), 3.94 (s, 4H), 3.78 (s, 3H), 3.71 (s, 3H), 3.66 (s, 6H), 3.48 (s, 4H), 1.41 (s, 9H). ^13^C NMR (100 MHz, CDCl_3_) δ 165.60, 158.06, 153.41, 152.53, 136.93, 135.80, 129.61, 128.47, 112.65, 104.95, 79.63, 59.95, 55.20, 54.26, 52.09, 39.98, 27.34. HRMS calculated for C_29_H_40_N_3_O_7_S_2_, [M + H]^+^ *m/z*: 606.2308, found: 606.2316.

#### 2-((4-Methoxybenzyl)(3,4,5-trimethoxyphenyl)amino)-2-oxoethyl-4-methylpiperazine-1-carbodithioate (C3)

White powder, yield, 64.5%, m.p:40–42 °C. ^1^H NMR (400 MHz, CDCl_3_) δ 7.10 (d, *J* = 8.6 Hz, 2H), 6.73 (d, *J* = 8.6 Hz, 2H), 6.26 (s, 2H), 4.74 (s, 2H), 4.23 (s, 2H), 3.94 (s, 4H), 3.78 (s, 3H), 3.71 (s, 3H), 3.66 (s, 6H), 2.50 − 2.38 (m, 4H), 2.26 (s, 3H). ^13^C NMR (100 MHz, CDCl_3_) δ 195.13, 165.70, 158.04, 152.51, 136.89, 135.86, 129.60, 128.54, 112.64, 104.96, 59.94, 55.19, 54.26, 53.24, 52.04, 44.51, 40.04, 28.26. HRMS calculated for C_25_H_34_N_3_O_5_S_2_, [M + H]^+^ *m/z*: 520.1940, found: 520.1949.

#### 3-((4-Methoxybenzyl)(3,4,5-trimethoxyphenyl)amino)-3-oxopropyl-4-acetylpiperazine-1-carbodithioate (C4)

White powder, yield, 52.3%, m.p:46–48 °C. ^1^H NMR (400 MHz, CDCl_3_) δ 7.08 (d, *J* = 8.6 Hz, 2H), 6.74 (d, *J* = 8.6 Hz, 2H), 6.04 (s, 2H), 4.70 (s, 2H), 3.92 (d, *J* = 25.2 Hz, 4H), 3.78 (s, 3H), 3.71 (s, 3H), 3.64 (d, *J* = 8.1 Hz, 8H), 3.59 − 3.43 (m, 4H), 2.52 (t, *J* = 7.0 Hz, 2H), 2.06 (s, 3H). ^13^C NMR (100 MHz, CDCl_3_) δ 169.82, 158.03, 152.52, 136.72, 136.18, 129.57, 128.75, 112.65, 104.87, 59.94, 55.18, 54.27, 51.39, 44.17, 39.54, 32.96, 31.53, 28.26, 20.34. HRMS calculated for C_27_H_36_N_3_O_6_S_2_, [M + H]^+^ *m/z*: 562.2046, found: 562.2052.

#### 4-((4-Methoxybenzyl)(3,4,5-trimethoxyphenyl)amino)-4-oxobutyl-4-methylpiperazine-1-carbodithioate (C5)

White powder, yield, 78.5%, m.p:86–88 °C. ^1^H NMR (400 MHz, CDCl_3_) δ 7.07 (d, *J* = 8.5 Hz, 2H), 6.73 (d, *J* = 8.5 Hz, 2H), 6.05 (s, 2H), 4.69 (s, 2H), 4.25 (s, 2H), 3.98 − 3.79 (m, 2H), 3.78 (s, 3H), 3.71 (s, 3H), 3.64 (s, 6H), 3.21 (t, *J* = 7.3 Hz, 2H), 2.39 (s, 4H), 2.25 (s, 3H), 2.17 (t, *J* = 7.2 Hz, 2H), 2.02 − 1.85 (m, 2H). ^13^C NMR (100 MHz, CDCl_3_) δ 195.90, 170.78, 157.96, 152.46, 136.67, 136.58, 129.51, 128.97, 112.64, 104.81, 59.93, 55.17, 54.26 53.34, 51.26, 44.59, 35.47, 32.28, 23.83. HRMS calculated for C_27_H_38_N_3_O_5_S_2_, [M + H]^+^ m/z: 548.2253, found: 548.2258.

#### 3-((4-Methoxybenzyl)(3,4,5-trimethoxyphenyl)amino)-3-oxopropyl-4-methylpiperazine-1-carbodithioate (C6)

White powder, yield, 63.1%, m.p:122–124 °C. ^1^H NMR (400 MHz, CDCl_3_) δ 7.08 (d, *J* = 8.6 Hz, 2H), 6.73 (d, *J* = 8.6 Hz, 2H), 6.04 (s, 2H), 4.70 (s, 2H), 4.23 (s, 2H), 3.88 (m, 2H), 3.77 (s, 3H), 3.71 (s, 3H), 3.63 (s, 6H), 3.52 (t, *J* = 7.0 Hz, 2H), 2.52 (t, *J* = 7.0 Hz, 2H), 2.46 − 2.30 (m, 4H), 2.24 (s, 3H). ^13^C NMR (100 MHz, CDCl_3_) δ 195.99, 169.97, 157.99, 152.49, 136.67, 136.24, 129.55, 128.83, 112.63, 104.87, 59.94, 55.16, 54.26, 53.36, 51.36, 44.60, 33.09, 31.48. HRMS calculated for C_26_H_36_N_3_O_5_S_2_, [M + H]^+^ *m/z*: 534.2096, found: 534.2102.

#### 3-((4-Methoxybenzyl)(3,4,5-trimethoxyphenyl)amino)-3-oxopropyl-4-ethylpiperazine-1-carbodithioate (C7)

White powder, yield, 50.6%, m.p:124–126 °C. ^1^H NMR (400 MHz, CDCl_3_) δ 7.08 (d, *J* = 8.6 Hz, 2H), 6.73 (d, *J* = 8.6 Hz, 2H), 6.04 (s, 2H), 4.70 (s, 2H), 4.23 (s, 2H), 3.88 (s, 2H), 3.77 (s, 3H), 3.71 (s, 3H), 3.63 (s, 6H), 3.52 (t, *J* = 7.0 Hz, 2H), 2.52 (t, *J* = 7.0 Hz, 2H), 2.42 (m, 4H), 2.37 (q, *J* = 7.2 Hz, 2H), 1.02 (t, *J* = 7.2 Hz, 3H). ^13^C NMR (100 MHz, CDCl_3_) δ 195.77, 169.98, 157.99, 152.48, 136.66, 136.24, 129.55, 128.84, 112.63, 104.87, 59.94, 55.16, 54.25, 51.36, 51.12, 50.88, 33.10, 31.47, 10.93. HRMS calculated for C_27_H_38_N_3_O_5_S_2_, [M + H]^+^ *m/z*: 548.2253, found: 548.2259.

#### 2-((4-Methoxybenzyl)(3,4,5-trimethoxyphenyl)amino)-2-oxoethyl-4-ethylpiperazine-1-carbodithioate (C8)

White powder, yield, 73.1%, m.p:41–43 °C. ^1^H NMR (400 MHz, CDCl_3_) δ 7.10 (d, *J* = 8.6 Hz, 2H), 6.73 (d, *J* = 8.6 Hz, 2H), 6.26 (s, 2H), 4.74 (s, 2H), 4.27 (s, 2H), 3.94 (s, 4H), 3.78 (s, 3H), 3.71 (s, 3H), 3.66 (s, 6H), 2.53 (m, 4H), 2.44 (q, *J* = 7.2 Hz, 2H), 1.05 (t, *J* = 7.2 Hz, 3H). ^13^C NMR (100 MHz, CDCl_3_) δ 195.03, 165.69, 158.04, 152.51, 136.90, 135.85, 129.60, 128.52, 112.64, 104.96, 59.94, 55.19, 54.26, 52.05, 50.83, 40.03, 28.26, 10.61. HRMS calculated for C_26_H_36_N_3_O_5_S_2_, [M + H]^+^ *m/z*: 534.2096, found: 534.2104.

#### 4-((4-Methoxybenzyl)(3,4,5-trimethoxyphenyl)amino)-4-oxobutyl-4-ethylpiperazine-1-carbodithioate (C9)

Yellow liquid, yield, 52.9%. ^1^H NMR (400 MHz, CDCl_3_) δ 7.07 (d, *J* = 8.6 Hz, 2H), 6.73 (d, *J* = 8.6 Hz, 2H), 6.05 (s, 2H), 4.69 (s, 2H), 4.26 (s, 2H), 3.85 (s, 2H), 3.78 (s, 3H), 3.71 (s, 3H), 3.64 (s, 6H), 3.21 (t, *J* = 7.4 Hz, 2H), 2.42 (s, 4H), 2.38 (m, 2H), 2.17 (t, *J* = 7.2 Hz, 2H), 1.99 (m, 2H), 1.03 (t, *J* = 7.2 Hz, 3H). ^13^C NMR (100 MHz, CDCl_3_) δ 195.71, 170.78, 157.96, 152.47, 136.67, 129.51, 128.97, 112.65, 104.81, 59.94, 55.17, 54.26, 51.26, 51.08, 50.87, 35.45, 32.28, 23.84, 10.88. HRMS calculated for C_28_H_40_N_3_O_5_S_2_, [M + H]^+^ *m/z*: 562.2409, found: 562.2413.

#### 2-((4-Methoxybenzyl)(3,4,5-trimethoxyphenyl)amino)-2-oxoethyl-pyrrolidine-1-carbodithioate (C10)

White powder, yield, 80.2%, m.p:150–152 °C. ^1^H NMR (400 MHz, CDCl_3_) δ 7.11 (d, *J* = 8.5 Hz, 2H), 6.73 (d, *J* = 8.5 Hz, 2H), 6.27 (s, 2H), 4.75 (s, 2H), 3.94 (s, 2H), 3.83 (t, *J* = 7.0 Hz, 2H), 3.78 (s, 3H), 3.71 (s, 3H), 3.67 (s, 6H), 3.65 (d, *J* = 7.1 Hz, 2H), 2.07 − 1.95 (m, 2H), 1.90 (p, *J* = 6.7 Hz, 2H). ^13^C NMR (100 MHz, CDCl_3_) δ 190.78, 165.92, 158.02, 152.49, 136.86, 135.91, 129.59, 128.58, 112.64, 105.01, 59.93, 55.20, 54.25, 54.23, 52.07, 49.74, 39.52, 25.13, 23.32. HRMS calculated for C_24_H_31_N_2_O_5_S_2_, [M + H]^+^ *m/z*: 491.1674, found: 491.1678.

#### 2-((4-Methoxybenzyl)(3,4,5-trimethoxyphenyl)amino)-2-oxoethylpiperidine-1-carbodithioate (C11)

Yellow liquid, yield, 52.9%. ^1^H NMR (400 MHz, CDCl_3_) δ 7.07 (d, *J* = 8.6 Hz, 2H), 6.73 (d, *J* = 8.6 Hz, 2H), 6.05 (s, 2H), 4.69 (s, 2H), 4.26 (s, 2H), 3.85 (s, 2H), 3.78 (s, 3H), 3.71 (s, 3H), 3.64 (s, 6H), 3.21 (t, *J* = 7.4 Hz, 2H), 2.42 (s, 4H), 2.38 (m, 2H), 2.17 (t, *J* = 7.2 Hz, 2H), 1.99 (m, 2H), 1.03 (t, *J* = 7.2 Hz, 3H). ^13^C NMR (100 MHz, CDCl_3_) δ 195.71, 170.78, 157.96, 152.47, 136.67, 129.51, 128.97, 112.65, 104.81, 59.94, 55.17, 54.26, 51.26, 51.08, 50.87, 35.45, 32.28, 23.84, 10.88. HRMS calculated for C_28_H_40_N_3_O_5_S_2_, [M + H]^+^ *m/z*: 562.2409, found: 562.2413.

#### 2-((4-Methoxybenzyl)(3,4,5-trimethoxyphenyl)amino)-2-oxoethyl-3-methylpiperidine-1-carbodithioate (C12)

White powder, yield, 48.6%, m.p:113–115 °C. ^1^H NMR (400 MHz, CDCl_3_) δ 7.11 (d, *J* = 8.5 Hz, 2H), 6.73 (d, *J* = 8.5 Hz, 2H), 6.27 (s, 2H), 5.22 (d, *J* = 10.7 Hz, 1H), 4.75 (d, *J* = 9.0 Hz, 2H), 4.45 (dd, 1H), 4.12 (m, 2H), 3.78 (s, 3H), 3.71 (s, 3H), 3.66 (s, 6H), 3.09 (s, 1H), 2.77 (m, 1H), 1.79 (d, *J* = 13.1 Hz, 1H), 1.68 (s, 2H), 1.60 (m, 1H), 1.18 (m, 1H), 0.88 (d, *J* = 6.5 Hz, 3H). ^13^C NMR (100 MHz, CDCl_3_) δ 193.67, 165.91, 158.01, 152.47, 136.83, 135.93, 129.59, 128.61, 112.62, 104.97, 59.93, 55.18, 54.25, 52.01, 40.10, 31.79, 17.74. HRMS calculated for C_26_H_35_N_2_O_5_S_2_, [M + H]^+^ *m/z*: 519.1987, found: 519.1996.

#### 2-((4-Methoxybenzyl)(3,4,5-trimethoxyphenyl)amino)-2-oxoethyl-morpholine-4-carbodithioate (C13)

White powder, yield, 70.1%, m.p:163–165 °C. ^1^H NMR (400 MHz, CDCl_3_) δ 7.11 (d, *J* = 8.5 Hz, 2H), 6.74 (d, *J* = 8.6 Hz, 2H), 6.26 (s, 2H), 4.74 (s, 2H), 4.21 (s, 2H), 3.95 (s, 4H), 3.78 (s, 3H), 3.71 (s, 3H), 3.69 (m, 4H), 3.67 (s, 6H). ^13^C NMR (100 MHz, CDCl_3_) δ 195.68, 165.57, 158.05, 152.53, 136.91, 135.82, 129.61, 128.49, 112.64, 104.94, 65.17, 59.94, 55.19, 54.25, 52.07, 39.86. HRMS calculated for C_24_H_31_N_2_O_6_S_2_, [M + H]^+^ *m/z*: 507.1624, found: 507.1629.

#### 2-((4-Methoxybenzyl)(3,4,5-trimethoxyphenyl)amino)-2-oxoethyl-3,5-dimethylpiperidine-1-carbodithioate (C14)

White powder, yield, 51.3%, m.p:128–130 °C. ^1^H NMR (400 MHz, CDCl_3_) δ 7.11 (d, *J* = 8.5 Hz, 2H), 6.73 (d, *J* = 8.5 Hz, 2H), 6.26 (s, 2H), 5.41 (d, *J* = 10.7 Hz, 1H), 4.87 − 4.60 (m, 2H), 4.52 (d, *J* = 12.4 Hz, 1H), 4.11 − 3.83 (m, 2H), 3.78 (s, 3H), 3.71 (s, 3H), 3.66 (s, 6H), 2.61 (t, *J* = 12.0 Hz, 1H), 2.40 (t, *J* = 11.8 Hz, 1H), 1.79 (d, *J* = 13.0 Hz, 1H), 1.74 − 1.58 (m, 2H), 0.88 (s, 3H), 0.86 (s, 3H), 0.79 (m, 1H). ^13^C NMR (100 MHz, CDCl_3_) δ 193.48, 165.91, 158.01, 152.47, 136.83, 135.92, 129.59, 128.59, 112.62, 104.96, 59.93, 57.89, 56.34, 55.18, 54.25, 52.01, 41.12, 40.14, 30.74, 29.86, 17.81. HRMS calculated for C_27_H_37_N_2_O_5_S_2_, [M + H]^+^ *m/z*: 533.2144, found: 533.2148.

#### 2-((4-Methoxybenzyl)(3,4,5-trimethoxyphenyl)amino)-2-oxoethyl-2,6-dimethylpiperidine-1-carbodithioate (C15)

White powder, yield, 76.2%, m.p:124–126 °C. ^1^H NMR (400 MHz, CDCl_3_) δ 7.12 (d, *J* = 8.5 Hz, 2H), 6.73 (d, *J* = 8.5 Hz, 2H), 6.27 (s, 2H), 5.75 − 5.50 (m, 1H), 5.00 − 4.82 (m, 1H), 4.82 − 4.66 (m, 2H), 4.10 (s, 1H), 3.81 (s, 1H), 3.77 (s, 3H), 3.71 (s, 3H), 3.67 (s, 6H), 1.83 − 1.48 (m, 6H), 1.31 (d, *J* = 7.0 Hz, 3H), 1.23 (d, *J* = 7.1 Hz, 3H). ^13^C NMR (100 MHz, CDCl_3_) δ 194.73, 166.04, 158.00, 152.47, 136.81, 136.01, 129.58, 128.66, 112.62, 104.97, 59.93, 55.18, 54.25, 53.12, 52.25, 51.98, 39.85, 29.32, 29.11, 18.87, 17.59, 12.81. HRMS calculated for C_27_H_37_N_2_O_5_S_2_, [M + H]^+^ *m/z*: 533.2144, found: 533.2150.

### Biology

#### MTT assay

Tubulin and NEDDylation have been promising antitumor targets and play vital roles in diverse cancers, such as liver cancer, prostate cancer, and breast cancer^15^. Therefore, liver cancer cell line (HepG2), prostate cancer cell line (PC3), breast cancer cell line (MCF7), and normal human liver cell line (L-02) were selected to evaluate their antiproliferative activity^16^^,^^17^. HepG2, PC3, MCF7, and L-02 cell lines were cultured at 37 °C in the RPMI1640 medium (Shanghai Yuanye Biotechnology Co., LTD, shanghai, china). Cancer cell lines were seeded into the 48-well plates for 24 h and 10 000 cells were seeded into each well. Compounds were dissolved by dimethyl sulphoxide and added into above plates. Then, all plates were incubated for 72 h at 37 °C and MTT solution (Shanghai Yuanye Biotechnology Co., LTD, shanghai, china) was added. After the incubation of MTT solution for 2 h, 100 μL dimethyl sulphoxide was added to each well and shook for 15 min. The absorbance of each well was determined by a microplate reader (Thermo Fisher Scientific, Waltham, MA) at 475 nm.

#### Tubulin polymerisation assay in vitro

An amount of 1 mM ATP, 10.2% glycerol, 0.5 mM magnesium chloride, 1 mM EGTA, and 80 mM PIPES were mixed with tubulin solution (5.6 mg/mL) to prepare the PEM buffer^18^. Then, compounds were dissolved using dimethyl sulphoxide and added into the PEM buffer. Finally, the system was monitored every minute at 37 °C by a spectrophotometer (Thermo Fisher Scientific, Waltham, MA). The absorbance was 420 nm and the excitation wavelength was 340 nm. The concentration of colchicine (3.5 nM) and methods of this assay were reported in our previous reference^19^.

#### Immunostaining assay

Human liver cancer cell line (HepG2) was seeded on a slice and cultured for 12 h^20^. Compound was dissolved using dimethyl sulphoxide and added into the slice. After the incubation for 48 h, the slice was fixed by 4% paraformaldehyde (Shanghai Yuanye Biotechnology Co., LTD, Shanghai, China) for 20 min. 0.1% BSA was added to block for 30 min and tubulin antibody (1:100) was incubated together for 12 h. The slice was washed and incubated with the secondary antibody. Finally, DAPI (Shanghai Yuanye Biotechnology Co., LTD, shanghai, china) was added to stain for three minutes.

#### NEDDylation assay in vitro

NEDDylation assay kit was purchased from Abcam (Cambridge, MA). Ubc12, NAE, NEDD8, and MgCl_2_ were added to prepare the NEDDylation solution. Different concentrations of target compound and ATP (0 mM or 1 mM) were added into the tubes with the NEDDylation solution. Above system was reacted at 37 °C for 1 h. Then, western blot was performed with anti-NEDD8 antibody to detect the Ubc12-NEDD8 band and Ubc12-(NEDD8)_2_ band.

#### Molecular docking

Molecular docking in this work was performed by the Autodock software (The Scripps Research Institute, San Diego,  CAA). The crystal structure of tubulin was 1SA0 and the crystal structure of E1 NAE was 3GZN. The formation of Grid for tubulin was *x* = 42.2, *y* = 52.9, *z* = −9.1. Docking store of **C11** with tubulin was −8.4 kcal/mol, and the store of **C14** with tubulin was −8.2 kcal/mol. The formation of Grid for E1 NAE was *x* = 10.1, *y* = 88.1, *z* = 44.5. Docking store of **C11** with E1 NAE was −9.1 kcal/mol. Both protein structures were removed the water and metal ions. After the docking, hydrophilic interaction, hydrophobic interaction, and hydrogen bonds were analysed between the target compound and proteins.

## Results and discussion

### Synthesis of compounds C1–C14

**Scheme 1.**
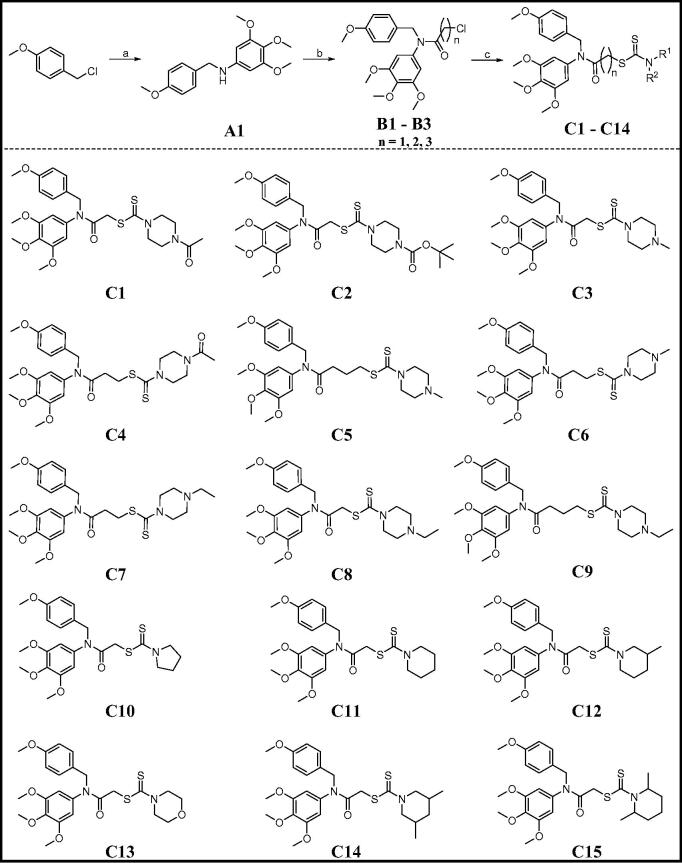
Reagents and conditions: (i) 3,4,5-Trimethoxyaniline, CH_2_Cl_2_, triethylamine, rt, 12 h; (ii) Acyl chlorides, CH_2_Cl_2_, rt, 2 h; (iii) CS_2_, Secondary amines, KOH, acetone, reflux, 7 h.

Synthetic route of target compounds **C1**–**C14** in this work was demonstrated in Scheme 1. 1-(Chloromethyl)-4-methoxybenzene and 3,4,5-trimethoxyaniline were reacted to perform the nucleophilic substitution^21^^,^^22^, and an acylation reaction was nextly started to obtain the amide intermediates **B1**–**B3**. These amide intermediates were reacted with carbon disulphide and secondary amines in the presence of potassium hydroxide to afford compounds **C1**–**C14**. All NMR analyses and of compounds **C1**–**C14** were illustrated in Supplemental data.

### Antiproliferative activity of compounds C1–C15

In order to develop novel antiproliferative agents, target compounds **C1**–**C15** were evaluated for their antiproliferative activity against HepG2 (human liver cancer), PC3 (human prostate cancer), and MCF7 (human breast cancer) cell lines by MTT assay. According to our reported reference, 5-fluorouracil was used as a control drug to determine the antiproliferative activity of amino dithiocarbamate analogues^23^. Thus, 5-fluorouracil was selected as the control drug in this work. In addition, colchicine as a tubulin polymerisation inhibitor and Gartanin as a NEDDylation inhibitor were also chosen as control agents.^24^ All inhibitory activity results against cancer cells for 72 h were listed in Table 1.

**Table 1.** Antiproliferative activity of compounds **A1** and **C1**–**C15**. 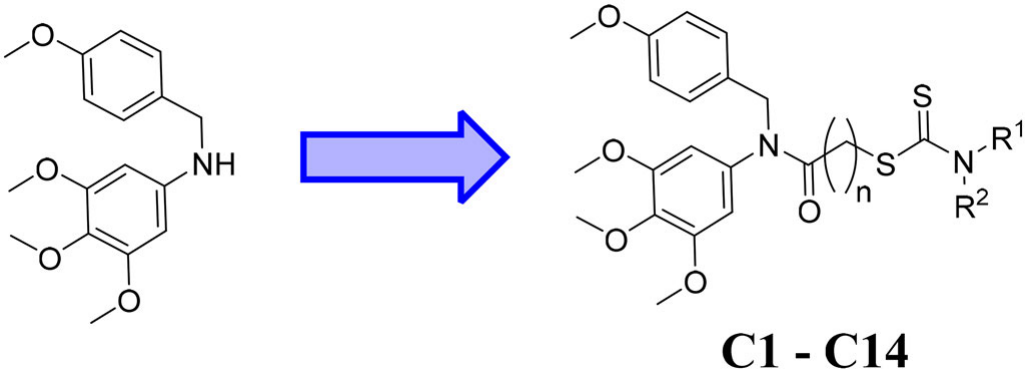

Compound	HepG2	PC3	MCF7
**A1**	> 30	> 30	> 30
**C1**	2.07 ± 0.14	2.96 ± 0.75	4.63 ± 0.91
**C2**	2.34 ± 0.12	3.37 ± 1.26	3.57 ± 0.63
**C3**	3.50 ± 0.37	3.42 ± 0.50	5.67 ± 0.24
**C4**	10.16 ± 1.27	15.37 ± 1.25	14.31 ± 1.27
**C5**	7.12 ± 0.10	15.94 ± 0.43	10.52 ± 0.47
**C6**	5.66 ± 0.15	14.13 ± 1.90	12.56 ± 0.52
**C7**	12.68 ± 0.81	17.62 ± 2.17	13.13 ± 0.81
**C8**	2.13 ± 0.13	2.92 ± 0.40	4.65 ± 0.84
**C9**	10.83 ± 0.71	12.57 ± 0.76	17.52 ± 0.48
**C10**	5.08 ± 0.51	7.89 ± 0.47	2.37 ± 0.24
**C11**	1.17 ± 0.26	2.48 ± 0.67	1.47 ± 0.18
**C12**	7.09 ± 0.33	6.24 ± 0.31	15.72 ± 1.63
**C13**	3.16 ± 0.21	3.37 ± 0.53	1.81 ± 0.29
**C14**	17.71 ± 0.33	14.46 ± 0.72	18.43 ± 0.21
**C15**	6.49 ± 0.89	9.46 ± 0.21	10.87 ± 1.07
**Colchicine**	5.73 ± 0.26	6.25 ± 0.76	8.13 ± 0.46
**Gartanin**	11.68 ± 0.52	9.74 ± 0.29	7.43 ± 0.81
**5-Fluorouracil**	14.17 ± 2.37	17.65 ± 1.24	18.21 ± 0.87

### Structure–activity relationship

The importance of the side chain containing an amino dithiocarbamate fragment was explored. Compound **A1** without the side chain displayed very weak activity against all three cell lines (IC_50_ > 30 μM). However, compounds **C1**–**C15** containing the side chain exhibited obviously inhibitory effects against these cancer cell lines with IC_50_ values from 1.17 μM to 18.43 μM. Especially, compound **C11** showed the most potent inhibitory activity against HepG2, PC3 and MCF7 cells with IC_50_ values of 1.17, 2.48, and 1.47 μM, respectively. Thses activity results indicated that the side chain played a significant role for antiproliferative activity.

Due to the importance of this side chain, compounds containing various side chains were designed and synthesised. We observed that the length of carbon linker between the amide group and dithiocarbamate fragment affected the inhibitory activity against all three cell lines. Compounds **C1**, **C3,** and **C8** with one carbon atom between the amide group and dithiocarbamate fragment displayed the good potency against HepG2, PC3, and MCF7 cells with IC_50_ values from 2.07 to 5.67 μM. Extension of the carbon linker length by one carbon or two carbons induced the decrease of antiproliferative activity (**C1** *VS* **C4**, **C3** *VS* **C5**, and **C8** *VS* **C9**).

To determine whether substituent groups on the piperazine ring and different secondary amines possessed the effects against inhibitory activity, compounds with a piperazine ring (**C1**–**C9**), a pyrrolidine ring (**C10**), a piperidine ring (**C11–C12** and **C14**–**C15**) and a morpholine ring (**C13**) were evaluated for their antiproliferative activity. Replacement of the pyrrolidine ring of compound **C10** with a morpholine ring (**C13**) led to an increase of the activity. However, changing the piperidine ring (compound **C11**) to a piperazine ring (compound **C3**) induced a decrease of the activity against HepG2, PC3, and MCF7 cells. All these results demonstrated that substituent groups on the piperazine ring and different secondary amines played important roles for the inhibitory activity. Compound **C11** was also tested for the potential cytotoxicity against L-02 (normal human liver cell line). **C11** demonstrated no cytotoxicity against L-02 (>20 μM), indicating that it had a good selectivity between liver cancer cells (HepG2) and normal liver cells (L-02).

### Tubulin polymerisation activity in vitro

**Figure 4.**
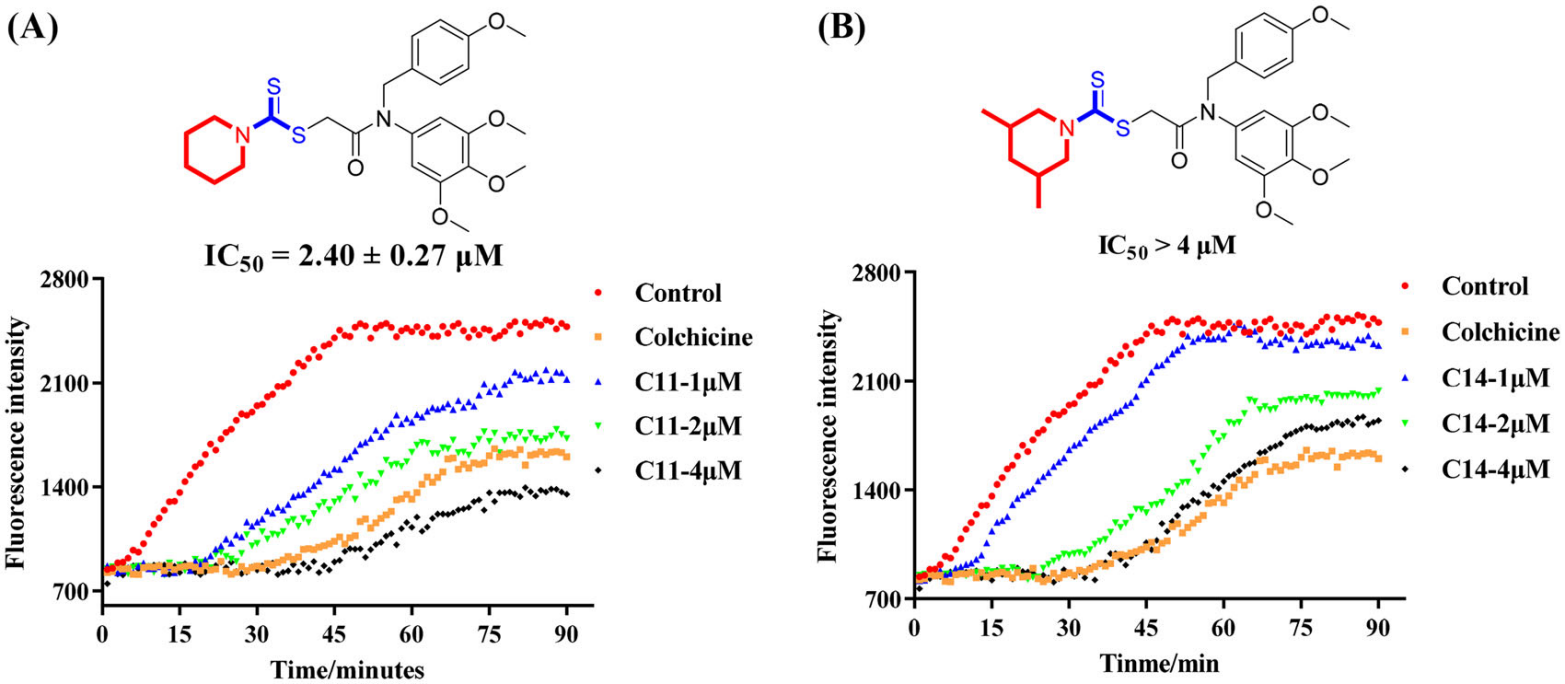
Tubulin polymerisation inhibitory activity of compound **C11** (**A**) and compound **C14** (**B**). The concentration of colchicine was 3.5 nM.

Because of the most potent inhibitory activity of compound **C11** against cancer cells, **C11** was selected to evaluate for its tubulin polymerisation inhibition activity. In addition, compound **C14** with the weak antiproliferative activity was also chosen for comparation with **C11**. Colchicine as a famous tubulin polymerisation inhibitor was used as the control drug in this assay. As shown in Figure 4(A), compound **C11** displayed the potent inhibitory activity against tubulin with an IC_50_ value of 2.40 μM. From inhibitory results of Figure 4(B), compound **C14** was also inhibited tubulin polymerisation in a concentration-dependent manner and its IC_50_ value was >4 μM. All these results indicated that compound **C11** was identified as a novel tubulin polymerisation inhibitor.

**Figure 5.**
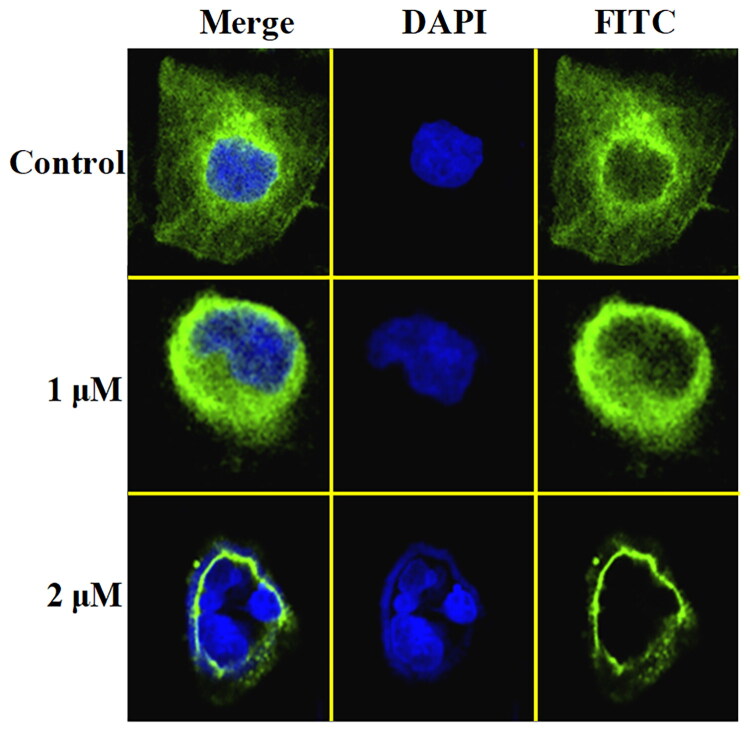
Imunofluorescence staining of compound **C11** against tubulin in HepG2 cells.

### Immunostaining of compound C11

Microtubules played important roles in the maintenance of basic cellular functions and cell shape, an immunofluorescence staining assay of compound **C11** was performed to demonstrate whether it could interfere the tubulin polymerisation in cancer cells. From results of immunofluorescence staining in Figure 5, compound **C11** at 1 μM moderately inhibited polymerisation of interphase microtubules in HepG2 cells. Importantly, compound **C11** at 2 μM displayed much stronger depolymerisation effects. All results of immunofluorescence staining illustrated that compound **C11** potently inhibited tubulin polymerisation in liver cancer HepG2 cells.

**Figure 6.**
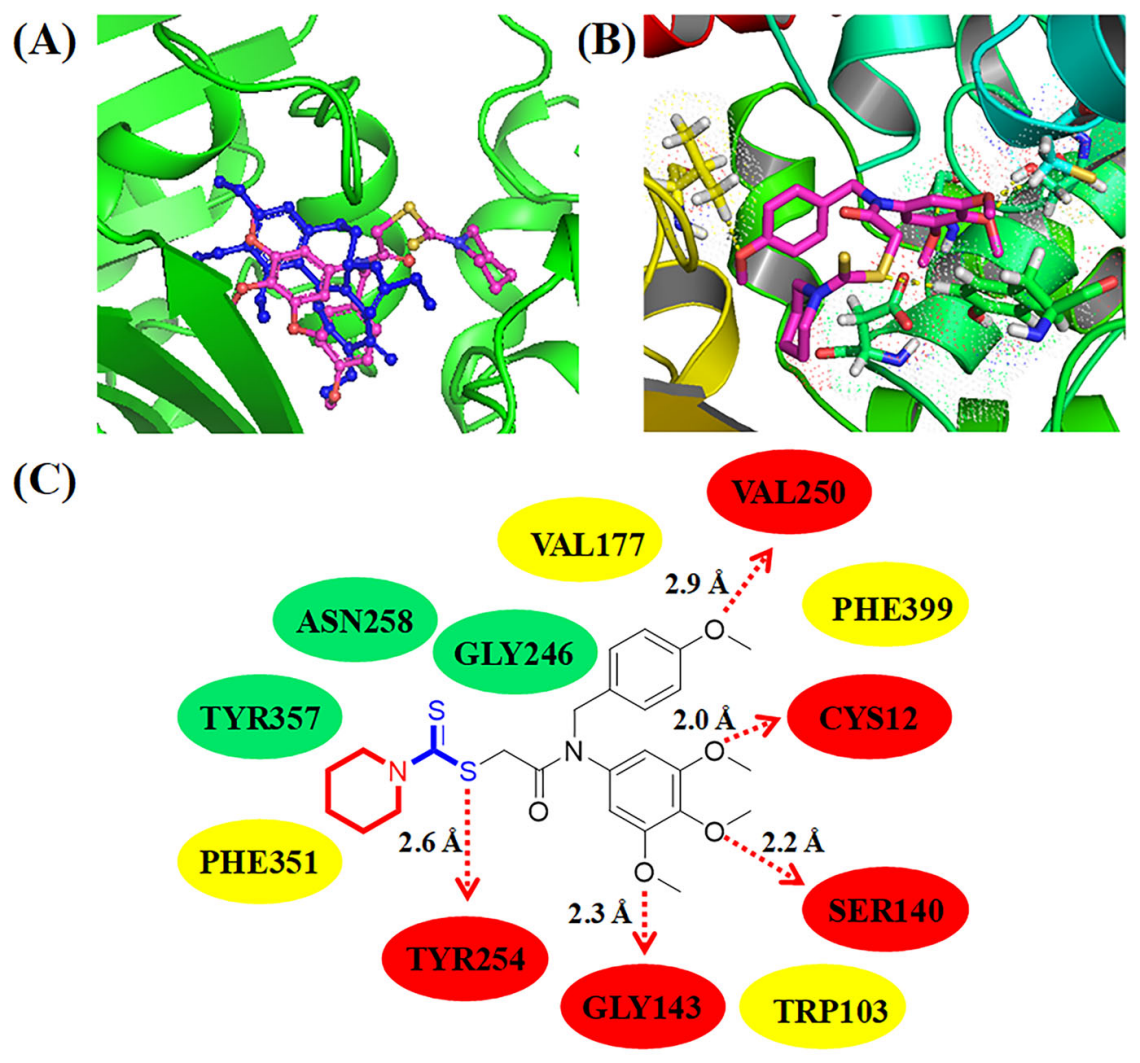
(A) Compound **C11** (pale red structure) docked into a similar pocket as colchicine (blue structure); (B) 3D binging modelling between **C11** and tubulin; (C) Hydrogen bonds, hydrophobic effects and hydrophilic effects of **C11** targeting to tubulin (PDB code: 1SA0).

### Molecular docking analysis of C11 and C14 binding to tubulin

In order to illustrate the potential binding effects between compound **C11** and tubulin, molecular docking analysis was performed using the autodock software. PDB code of tubulin was 1SA0 and its resolution was 3.58 Å. In Figure 6(A), **C11** (pale red structure) was binging to *β*-tubulin and docked into a similar pocket as colchicine (blue structure). As shown in Figure 6(B,C), three methoxy groups of 3,4,5-trimethoxy phenyl ring formed three hydrogen bonds with residues GLY143, SER140, and CYS12, respectively. The methoxy group of 4-methoxy phenyl ring formed one hydrogen bond with the residue VAL250. The dithiocarbamate group of compound **C11** formed a hydrogen bond with the residue TYR254. The hydrogen bond of TYR254 indicated that the side chain containing a dithiocarbamate group might play an important role for antiproliferative activity. Compound **C11** formed hydrophobic effects with residues VAL177, PHE399, TRP103, and PHE351, respectively. In addition, amino dithiopiperazine unit of compound **C11** also formed hydrophilic effects with residues TYR357, ASN258, and GLY246.

**Figure 7.**
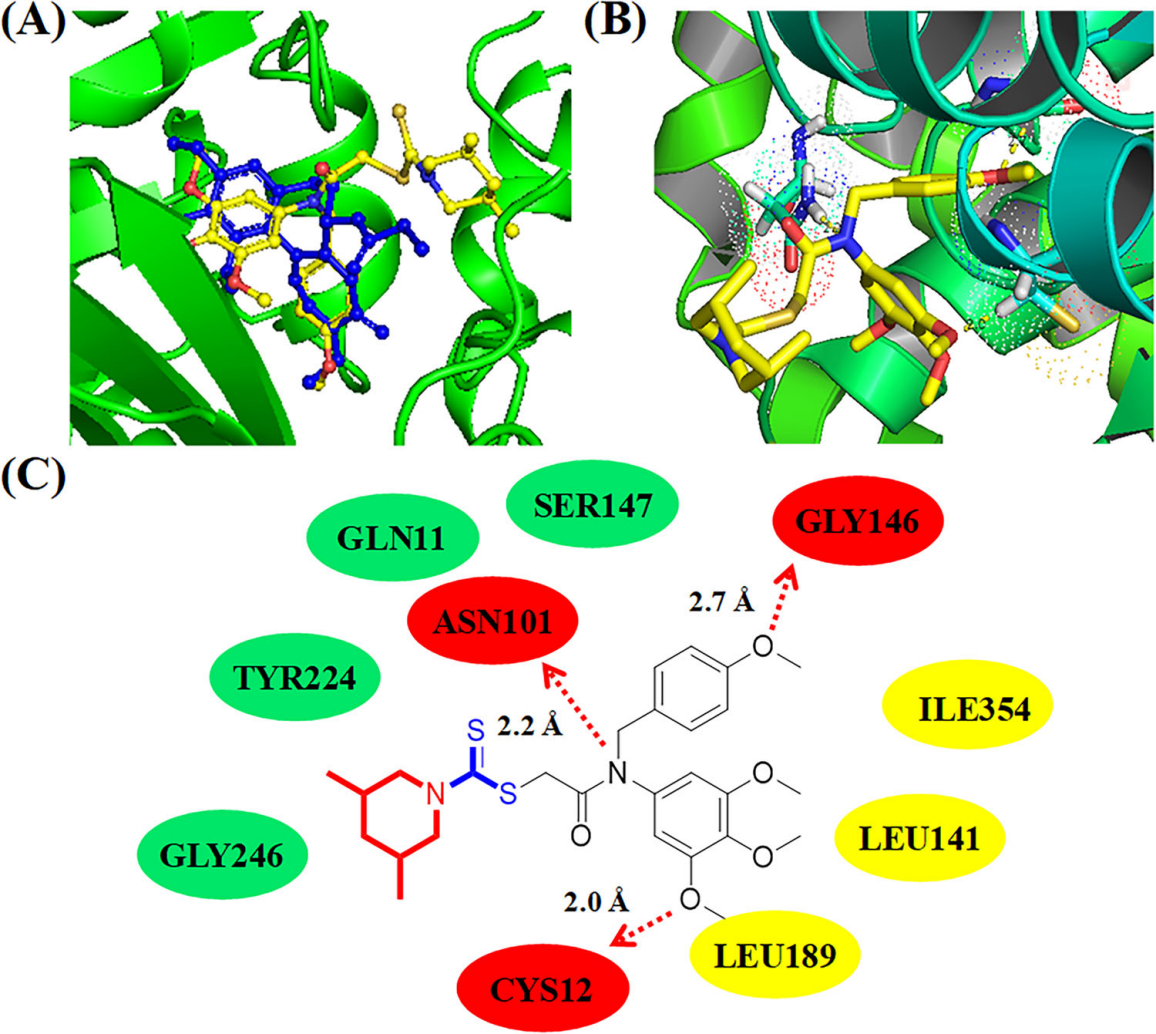
(A) Compound **C14** (yellow structure) docked into a similar pocket as colchicine (blue structure); (B) 3D binging modelling between **C14** and tubulin; (C) Hydrogen bonds, hydrophobic effects and hydrophilic effects of **C14** targeting to tubulin (PDB code: 1SA0).

With the comparation of compound **C11**, compound **C14** exhibited wore inhibitory activities against cancer cells and tubulin. To demonstrate the difference of its binding effects, molecular docking studies of compound **C14** targeting to tubulin was also explored. As shown in Figure 7, all methoxy groups of 3,4,5-trimethoxy phenyl ring only formed one hydrogen bond with residue CYS12. The methoxy group of 4-methoxy phenyl ring formed a hydrogen bond with the residue GLY146 and the amide group of **C14** formed a hydrogen bond with the residue ASN101. Compound **C14** formed hydrophobic effects with residues LEU141, LEU189, and ILE354. In addition, amino dithiopiperazine unit of compound **C14** formed hydrophilic effects with residues GLY246, TYR224, GLN11, and SER147. However, the dithiocarbamate group of compound **C14** could not form hydrogen bonds with residues of tubulin. All these results illustrated that compound **C11** were more potent than compound **C14** for the antiproliferative activity and tubulin polymerisation inhibition activity.

**Figure 8.**
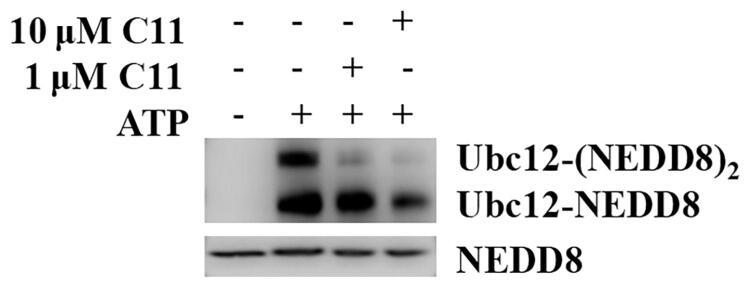
The inhibitory effects of compound **C11** on NEDDylation with or without ATP.

### Compound C11 inhibited NEDDylation in a ATP-dependent manner

NEDDylation as a biological process that affect the substrate’s activity, subcellular localisation, and conformation involves transfer of neural precursor cell-expressed developmentally downregulated 8 (NEDD8) in various cancers^25^. It is a three-step enzymatic cascade reaction by E1 NAE, NEDD8-conjugating enzymes E2 (Ubc12 or UBE2F), and NEDD8 E3 ligases^26^. To reveal the inhibitory effects of compound **C11** on NEDDylation, a NEDDylation assay was performed according to our reported reference^27^. As shown in Figure 8, there are no Ubc12-NEDD8 and Ubc12-(NEDD8)_2_ bands without the treatment of ATP. Furthermore, expression levels of Ubc12-NEDD8 and Ubc12-(NEDD8)_2_ were decreased with the treatment of compound **C11** in the presence of ATP. All these results obviously demonstrated that compound **C11** inhibited NEDDylation in a ATP-dependent manner and compound **C11** was a novel NEDDylation inhibitor.

**Figure 9.**
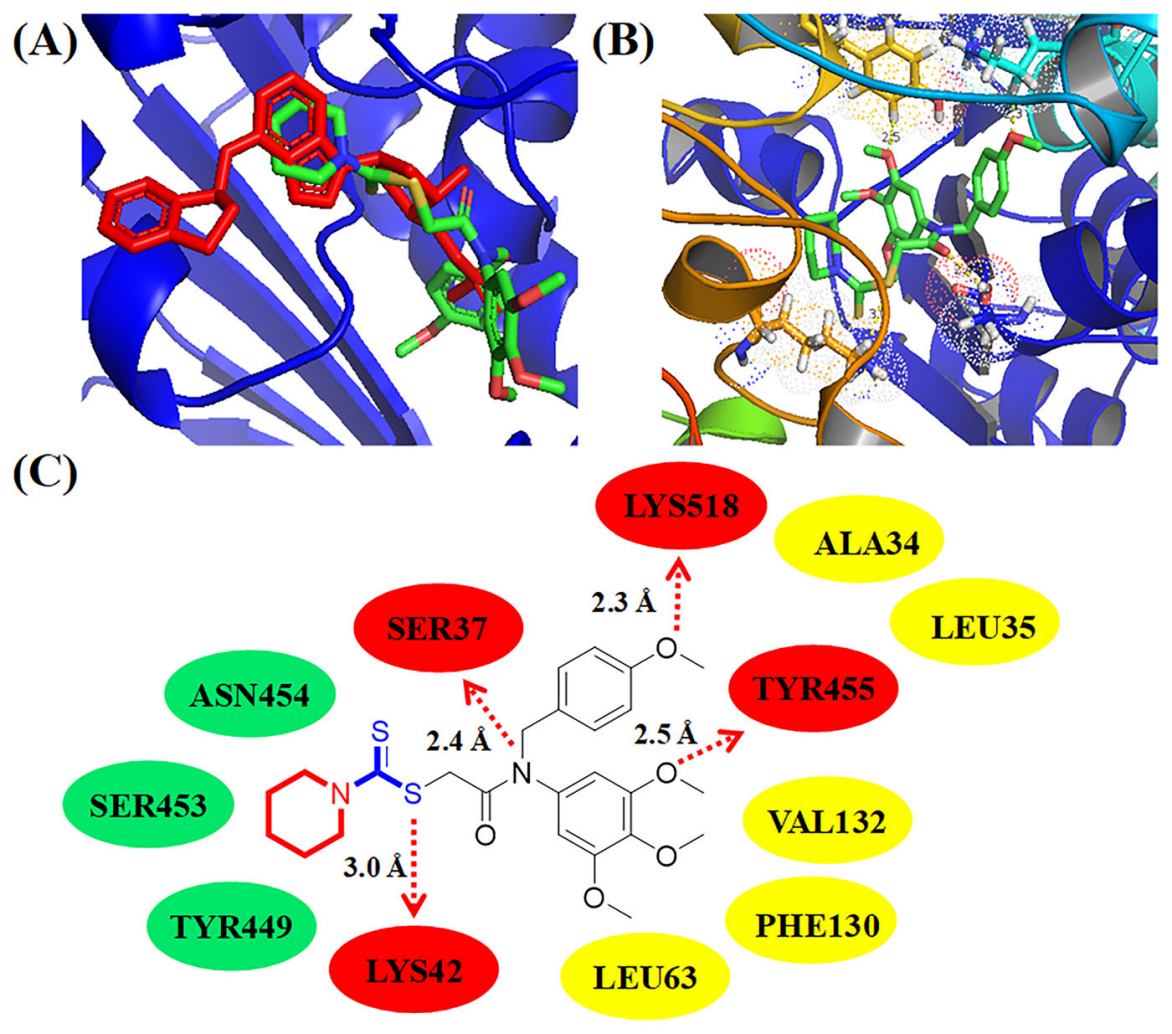
(A) Compound **C11** (green structure) docked into a similar pocket as B39 (red structure); (B) 3D binging modelling; (C) Hydrogen bonds, hydrophobic effects, and hydrophilic effects of **C11** targeting to E1 NEDD8-activating enzyme (PDB code: 3GZN).

### Molecular docking analysis of C11 binding to NAE from NEDDylation

To deeply study the potential interactions between compound **C11** and NEDDylation, molecular docking studies were also performed in this work. PDB code of NAE from NEDDylation was 3GZN and its resolution was 3.00 Å. In Figure 9(A), **C11** (green structure) docked into a similar pocket as B39 (red structure). As shown in Figure 9(B,C), a methoxy group of 3,4,5-trimethoxy phenyl ring formed the hydrogen bond with the residue TYR455. The methoxy group of 4-methoxy phenyl ring formed one hydrogen bond with the residue LYS518. Its side chain containing a dithiocarbamate group and an amide group o formed two hydrogen bonds with residues LYS42 and SER37, respectively. Compound **C11** formed hydrophobic effects with residues ALA34, LEU35, VAL132, PHE130, and LEU63, respectively. Furthermore, amino dithiopiperazine unit of compound **C11** also formed hydrophilic effects with residues ASN454, SER453, and TYR449. Based on results of molecular docking studies, it showed that compound **C11** could bind to NAE from NEDDylation.

## Conclusion

A series of trimethoxyphenyl analogues were synthesised and evaluated for their antiproliferative activity against HepG2, PC3, and MCF7 cells. Among all these derivatives, compound **C11** exhibited the most potent inhibitory activity against HepG2, PC3, and MCF7 cells with IC_50_ values of 1.17, 2.48, and 1.47 μM, respectively. In addition, **C11** showed the potent inhibitory activity against tubulin with an IC_50_ value of 2.40 μM and it potently inhibited tubulin polymerisation against liver cancer HepG2 cells. Furthermore, compound **C11** inhibited NEDDylation by a ATP-dependent manner. Methoxy groups and the dithiocarbamate group of compound **C11** could form hydrogen bonds with residues of tubulin and NAE protein from NEDDylation. Therefore, compound **C11** was a dual tubulin-NEDDylation inhibitor.

## Supplementary Material

Supplemental Material

## References

[1] Zhong C, Kayamori K, Koide S, Shinoda D, Oshima M, Nakajima-Takagi Y, Nagai Y, Mimura N, Lennox W, Sheedy J. Efficacy of tubulin polymerization inhibitor in myelodysplastic syndrome. Blood 2020;136(1):35–35. 10.1111/cas.14684 PMC7734154 33037737

[2] Hong Y, Zhu YY, He Q, Gu SX. Indole derivatives as tubulin polymerization inhibitors for the development of promising anticancer agents. Bioorg Med Chem 2021;55:116597. 34995858 10.1016/j.bmc.2021.116597

[3] Qi J, Dong H, Huang J, Zhang S, Niu L, Zhang Y, Wang J. Synthesis and biological evaluation of N-substituted 3-oxo-1,2,3,4-tetrahydro-quinoxaline-6-carboxylic acid derivatives as tubulin polymerization inhibitors. Eur J Med Chem 2018;143:8–20. 29172084 10.1016/j.ejmech.2017.08.018

[4] Yang H, An B, Li X, Zeng W. Evaluation of 4-phenylamino-substituted naphthalene-1,2-diones as tubulin polymerization inhibitors. Bioorg Med Chem Lett 2018;28(18):3057–3063. 30093295 10.1016/j.bmcl.2018.07.047

[5] Fu DJ, Yang JJ, Li P, Hou YH, Huang SN, Tippin MA, Pham V, Song L, Zi X, Xue WL, et al. Bioactive heterocycles containing a 3,4,5-trimethoxyphenyl fragment exerting potent antiproliferative activity through microtubule destabilization. Eur J Med Chem 2018;157:50–61. 30075402 10.1016/j.ejmech.2018.07.060

[6] Donthiboina K, Anchi P, Sri Ramya PV, Karri S, Srinivasulu G, Godugu C, Shankaraiah N, Kamal A. Synthesis of substituted biphenyl methylene indolinones as apoptosis inducers and tubulin polymerization inhibitors. Bioorg Chem 2019;86:210–223. 30716619 10.1016/j.bioorg.2019.01.063

[7] Liu W, He M, Li Y, Peng Z, Wang G. A review on synthetic chalcone derivatives as tubulin polymerisation inhibitors. J Enzyme Inhib Med Chem 2022;37(1):9–38. 34894980 10.1080/14756366.2021.1976772 PMC8667932

[8] Han S, Shin H, Oh JW, Oh YJ, Her NG, Nam DH. The protein neddylation inhibitor MLN4924 suppresses patient-derived glioblastoma cells via inhibition of ERK and AKT signaling. Cancers 2019;11(12):1849. 10.3390/cancers11121849 PMC6966592 31771104

[9] Yu Q, Jiang Y, Sun Y. Anticancer drug discovery by targeting cullin neddylation. Acta Pharm Sin B (5)2020;10:746–765. 32528826 10.1016/j.apsb.2019.09.005 PMC7276695

[10] Hammill JT, Bhasin D, Scott DC, Min J, Chen Y, Lu Y, Yang L, Kim HS, Connelly MC, Hammill C, et al. Discovery of an orally bioavailable inhibitor of defective in cullin neddylation 1 (DCN1)-mediated cullin neddylation. J Med Chem 2018;61(7):2694–2706. 29547693 10.1021/acs.jmedchem.7b01282 PMC5914176

[11] Fu DJ, Song J, Zhu T, Pang XJ, Wang SH, Zhang YB, Wu BW, Wang JW, Zi X, Zhang SY, et al. Discovery of novel tertiary amide derivatives as NEDDylation pathway activators to inhibit the tumor progression in vitro and in vivo. Eur J Med Chem 2020;192:112153. 32135407 10.1016/j.ejmech.2020.112153

[12] Song J, Liu Y, Yuan XY, Liu WB, Li YR, Yu GX, Tian XY, Zhang YB, Fu XJ, Zhang SY, et al. Discovery of 1,2,4-triazine dithiocarbamate derivatives as NEDDylation agonists to inhibit gastric cancers. Eur J Med Chem 2021;225:113801. 34455358 10.1016/j.ejmech.2021.113801

[13] Li Z, Ding J, Chen C, Chang J, Huang B, Geng Z, Wang Z. Dual-target cancer theranostic for glutathione S-transferase and hypoxia-inducible factor-1α inhibition. Chem Commun (Camb). 2017;53(92):12406–12409. 29112209 10.1039/c7cc08162f

[14] Shuai W, Wang G, Zhang Y, Bu F, Zhang S, Miller DD, Li W, Ouyang L, Wang Y. Recent Progress on tubulin inhibitors with dual targeting capabilities for cancer therapy. J Med Chem 2021;64(12):7963–7990. 34101463 10.1021/acs.jmedchem.1c00100

[15] Mosmann T. Rapid colorimetric assay for cellular growth and survival: application to proliferation and cytotoxicity assays. J Immunol Methods 1983;65(1–2):55–63. 6606682 10.1016/0022-1759(83)90303-4

[16] Liu YN, Wang JJ, Ji YT, Zhao GD, Tang LQ, Zhang CM, Guo XL, Liu ZP. Design, synthesis, and biological evaluation of 1-methyl-1,4-dihydroindeno[1,2-c]pyrazole analogues as potential anticancer agents targeting tubulin colchicine binding site. J Med Chem 2016;59(11):5341–5355. 27172319 10.1021/acs.jmedchem.6b00071

[17] Gai W, Peng Z, Liu CH, et al. Advances in cancer treatment by targeting the neddylation pathway. Front Cell Dev Biol 2021;9:653882. 33898451 10.3389/fcell.2021.653882 PMC8060460

[18] Quan YP, Cheng LP, Wang TC, Pang W, Wu FH, Huang JW. Molecular modeling study, synthesis and biological evaluation of combretastatin A-4 analogues as anticancer agents and tubulin inhibitors. MedChemComm 2018;9(2):316–327. 30108925 10.1039/c7md00416h PMC6083788

[19] Fu DJ, Li P, Wu BW, Cui XX, Zhao CB, Zhang SY. Molecular diversity of trimethoxyphenyl-1,2,3-triazole hybrids as novel colchicine site tubulin polymerization inhibitors. Eur J Med Chem 2019;165:309–322. 30690300 10.1016/j.ejmech.2019.01.033

[20] Fu DJ, Fu L, Liu YC, Wang JW, Wang YQ, Han BK, Li XR, Zhang C, Li F, Song J, et al. Structure-activity relationship studies of β-lactam-azide analogues as orally active antitumor agents targeting the tubulin colchicine site. Sci Rep 2017;7(1):12788. 28986548 10.1038/s41598-017-12912-4 PMC5630639

[21] Abulkhair HS, Elmeligie S, Ghiaty A, El‐Morsy A, Bayoumi AH, Ahmed HEA, El‐Adl K, Zayed MF, Hassan MH, Akl EN, et al. In vivo- and in silico-driven identification of novel synthetic quinoxalines as anticonvulsants and AMPA inhibitors. Arch Pharm 2021;354(5):2000449. 10.1002/ardp.202000449 33559320

[22] Abul-Khair H, Elmeligie S, Bayoumi A, et al. Synthesis and evaluation of some new (1,2,4) triazolo(4,3-a)quinoxalin-4(5H)-one derivatives as AMPA receptor antagonists. J Heterocycl Chem 2013;50:1202–1208.

[23] Fu DJ, Zhang L, Song J, Mao RW, Zhao RH, Liu YC, Hou YH, Li JH, Yang JJ, Jin CY, et al. Design and synthesis of formononetin-dithiocarbamate hybrids that inhibit growth and migration of PC-3 cells via MAPK/Wnt signaling pathways. Eur J Med Chem 2017;127:87–99. 28038329 10.1016/j.ejmech.2016.12.027 PMC5567725

[24] Pham V, Rendon R, Le VX, Tippin M, Fu DJ, Le TH, Miller M, Agredano E, Cedano J, Zi X, et al. Gartanin is a novel NEDDylation inhibitor for induction of Skp2 degradation, FBXW2 expression, and autophagy. Mol Carcinog 2020;59(2):193–201. 31782573 10.1002/mc.23140 PMC6946862

[25] Zhang Z, Heng Y, Cheng W, Pan Y, Ni S, Li H. Reactive oxygen species (ROS)-response nanomedicine through knocking down a novel therapeutic target NEDD8-conjugating enzyme UBC12 (UBE2M) in the treatment of liver cancer. Mater Des 2021;204:109648.

[26] Heo MJ, Kang SH, Kim YS, Lee JM, Yu J, Kim HR, Lim H, Kim KM, Jung J, Jeong LS, et al. UBC12-mediated SREBP-1 neddylation worsens metastatic tumor prognosis. Int J Cancer 2020;147(9):2550–2563. 32449166 10.1002/ijc.33113

[27] Fu DJ, Cui XX, Zhu T, Zhang YB, Hu YY, Zhang LR, Wang SH, Zhang SY. Discovery of novel indole derivatives that inhibit NEDDylation and MAPK pathways against gastric cancer MGC803 cells. Bioorg Chem 2021;107:104634. 33476867 10.1016/j.bioorg.2021.104634

