# Supplementary material for: Discovery of dual tubulin-NEDDylation inhibitors with antiproliferative activity
Source: J Enzyme Inhib Med Chem. 2022 Nov 4;38(1):166–75. doi: 10.1080/14756366.2022.2136173 (PMC9639481; doi:10.1080/14756366.2022.2136173)
Supplement: Supplemental Material [file IENZ_A_2136173_SM2320.pdf]

Discovery of dual tubulin-NEDDylation inhibitors with antiproliferative activity

Dong-Jun Fu, Ting Wang*

Beijing Research Institute of Chinese Medicine, Beijing University of Chinese Medicine, Beijing 100029, China

*Correspondence: wangting1973@sina.com (T.W.)

2-((4-methoxybenzyl)(3,4,5-trimethoxyphenyl)amino)-2-oxoethyl-4-acetylpiperazine-1-carbodithioate (C1)

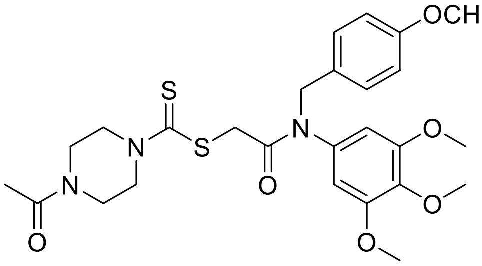

White powder, yield, 48.5 %, m.p:56~58 ºC. ^{1}H NMR (400 MHz, $CDCl_3$) δ 7.10 (d, *J* = 8.6 Hz, 2H), 6.74 (d, *J* = 8.6 Hz, 2H), 6.25 (s, 2H), 4.74 (s, 2H), 4.14 (s, 4H), 3.95 (s, 2H), 3.78 (s, 3H), 3.71 (s, 3H), 3.70 (m, 2H), 3.67 (s, 6H), 3.60 – 3.48 (m, 2H), 2.07 (s, 3H). ^{13}C NMR (100 MHz, $CDCl_3$) δ 168.33, 165.46, 158.08, 152.55, 136.96, 135.77, 129.60, 128.43, 112.66, 104.94, 59.95, 55.21, 54.26, 52.11, 44.15, 40.03, 29.92, 20.34. HRMS calculated for $C_{26}H_{34}N_3O_6S_2$, $[M+H]^+$ m/z: 548.1889, found: 548.1897.

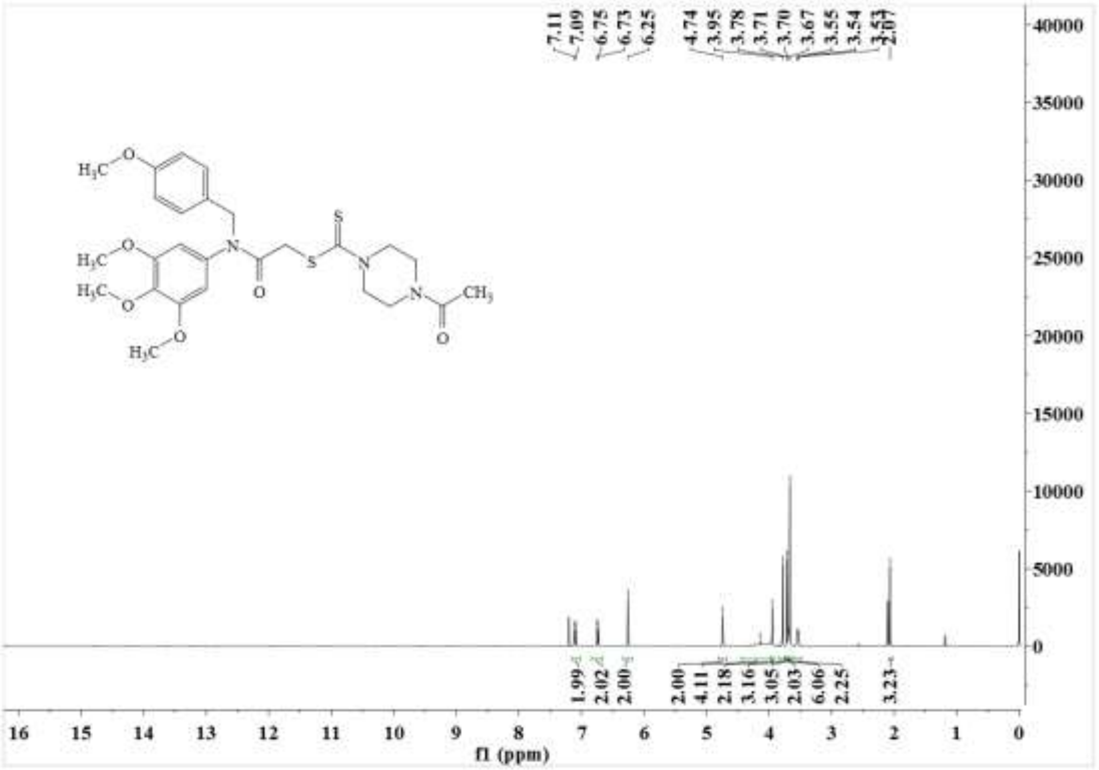

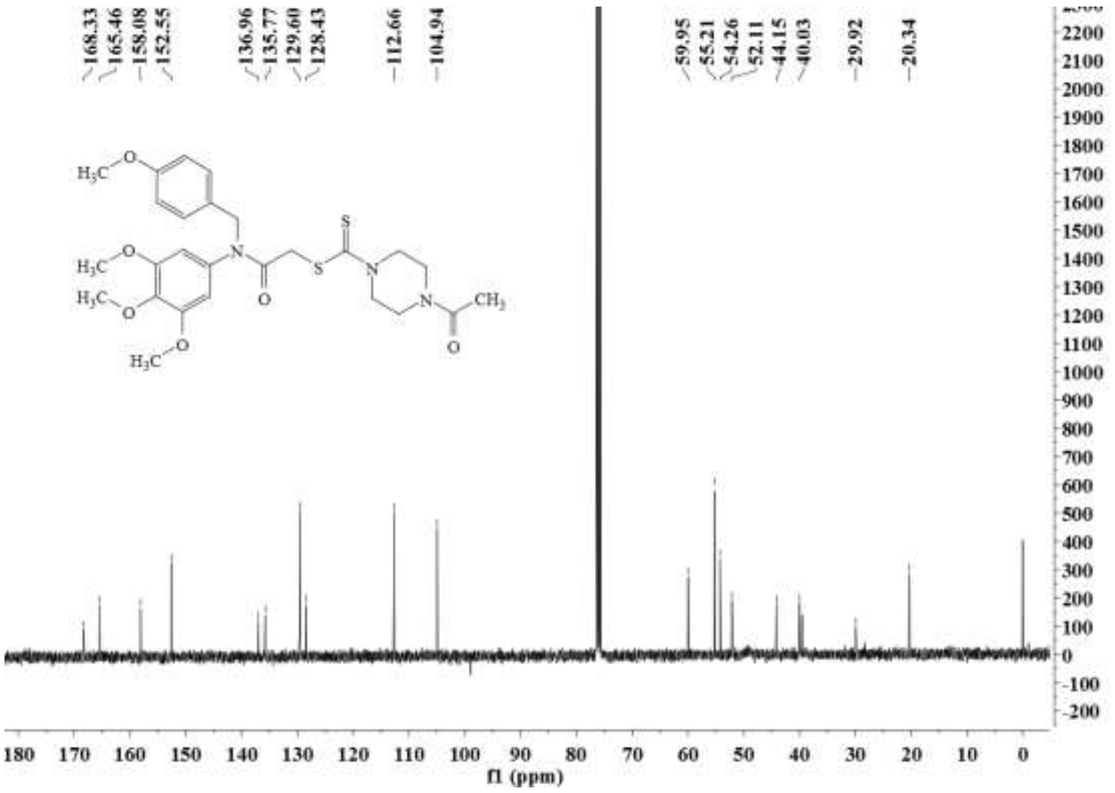

tert-butyl-4-(((2-((4-methoxybenzyl)(3,4,5-trimethoxyphenyl)amino)-2-oxoethyl)thio)carbonothioyl)piperazine-1-carboxylate (C2)

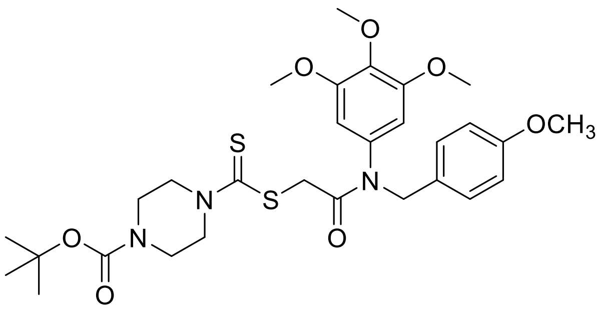

White powder, yield, 73.2 %, m.p:55~57 °C. ^{1}H NMR (400 MHz, $CDCl_3$) δ 7.10 (d, *J* = 8.6 Hz, 2H), 6.74 (d, *J* = 8.6 Hz, 2H), 6.25 (s, 2H), 4.74 (s, 2H), 4.17 (s, 2H), 3.94 (s, 4H), 3.78 (s, 3H), 3.71 (s, 3H), 3.66 (s, 6H), 3.48 (s, 4H), 1.41 (s, 9H). ^{13}C NMR (100 MHz, $CDCl_3$) δ 165.60, 158.06, 153.41, 152.53, 136.93, 135.80, 129.61, 128.47, 112.65, 104.95, 79.63, 59.95, 55.20, 54.26, 52.09, 39.98, 27.34. HRMS calculated for $C_{29}H_{40}N_3O_7S_2$, $[M+H]^+$ m/z: 606.2308, found: 606.2316.

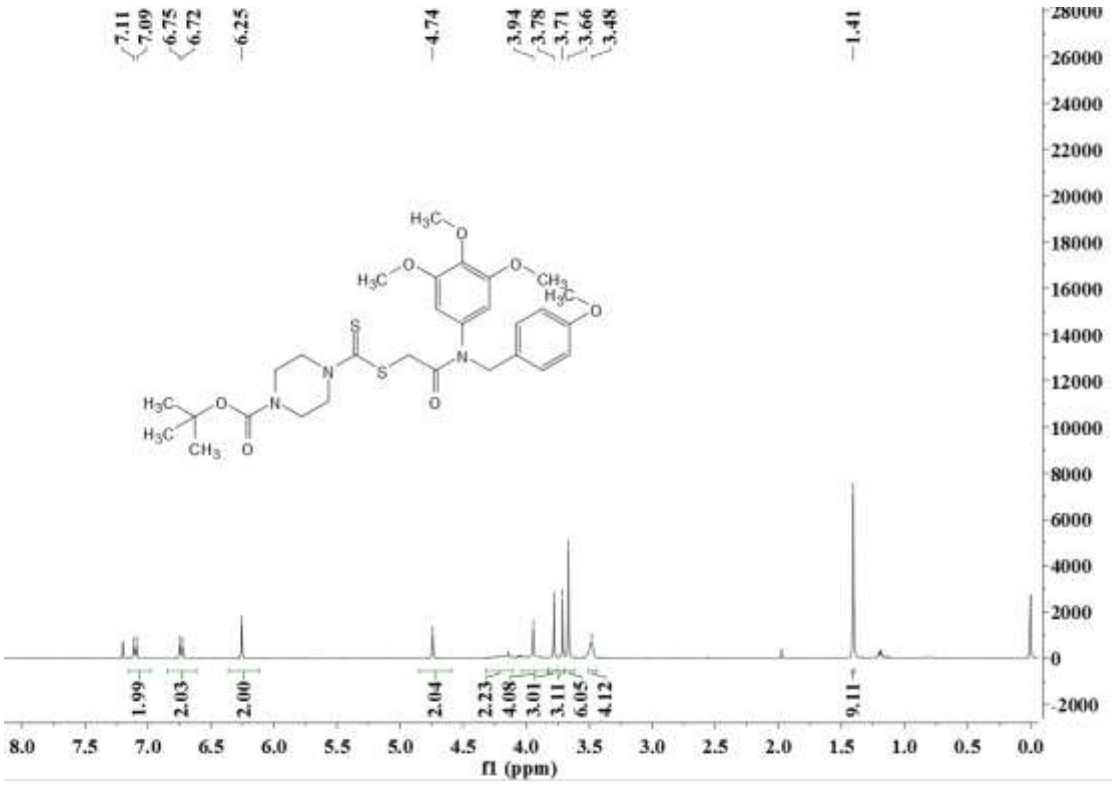

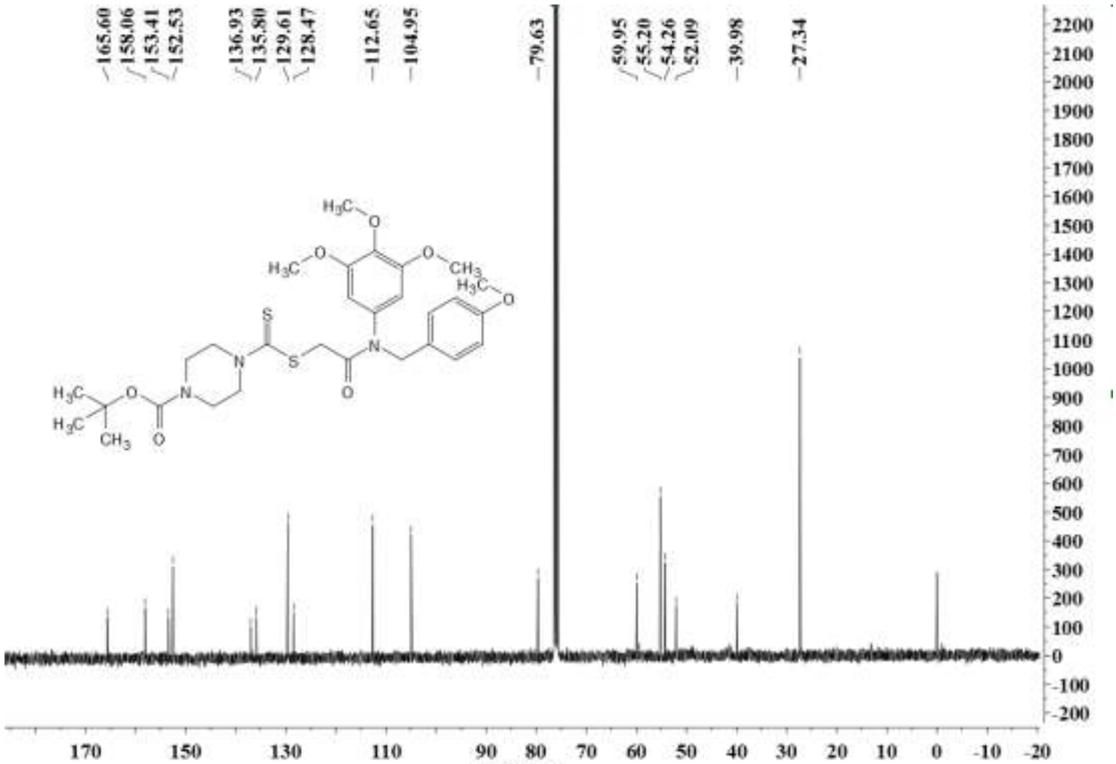

2-((4-methoxybenzyl)(3,4,5-trimethoxyphenyl)amino)-2-oxoethyl-4-methylpiperazine-1-carbodithioate (C3)

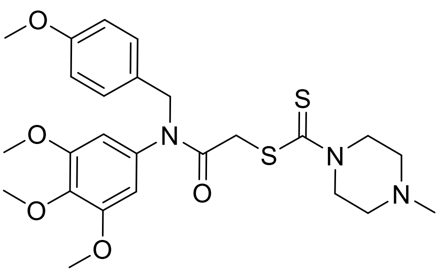

White powder, yield, 64.5 %, m.p:40~42 °C. ^{1}H NMR (400 MHz, $CDCl_3$) δ 7.10 (d, *J* = 8.6 Hz, 2H), 6.73 (d, *J* = 8.6 Hz, 2H), 6.26 (s, 2H), 4.74 (s, 2H), 4.23 (s, 2H), 3.94 (s, 4H), 3.78 (s, 3H), 3.71 (s, 3H), 3.66 (s, 6H), 2.50 – 2.38 (m, 4H), 2.26 (s, 3H). ^{13}C NMR (100 MHz, $CDCl_3$) δ 195.13, 165.70, 158.04, 152.51, 136.89, 135.86, 129.60, 128.54, 112.64, 104.96, 59.94, 55.19, 54.26, 53.24, 52.04, 44.51, 40.04, 28.26. HRMS calculated for $C_{25}H_{34}N_3O_5S_2$, $[M+H]^+$ m/z: 520.1940, found: 520.1949.

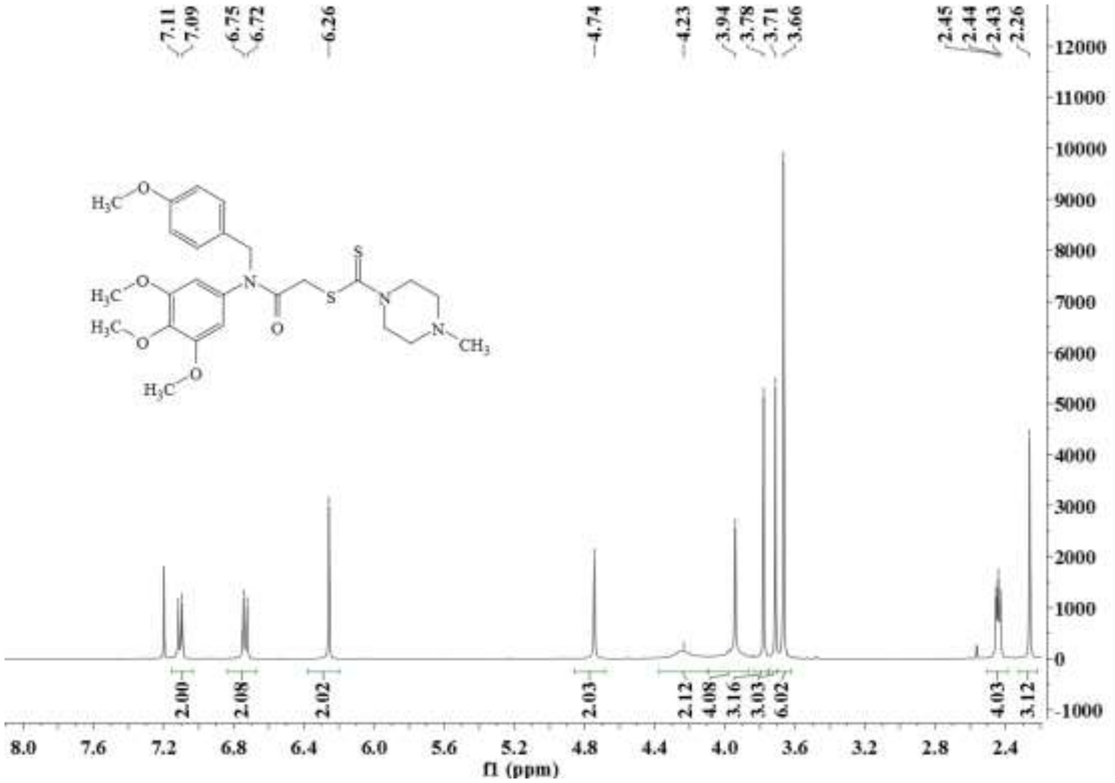

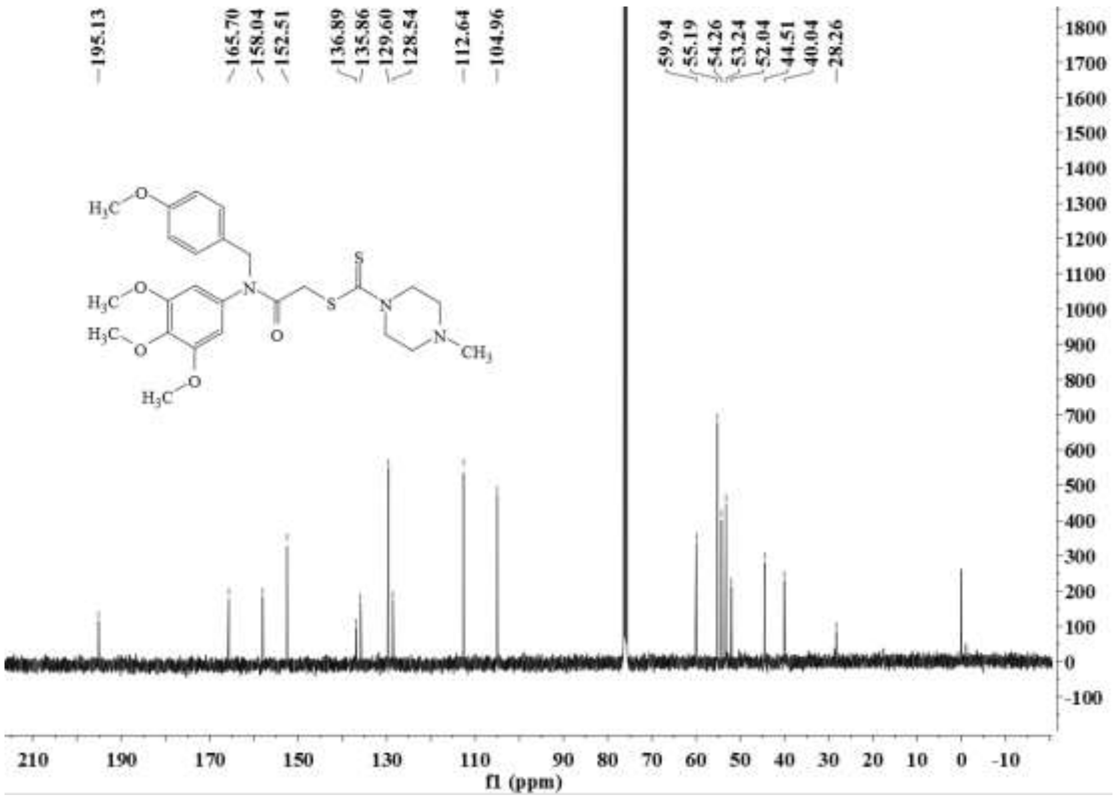

3-((4-methoxybenzyl)(3,4,5-trimethoxyphenyl)amino)-3-oxopropyl-4-acetylpiperazine-1-carbodithioate (C4)

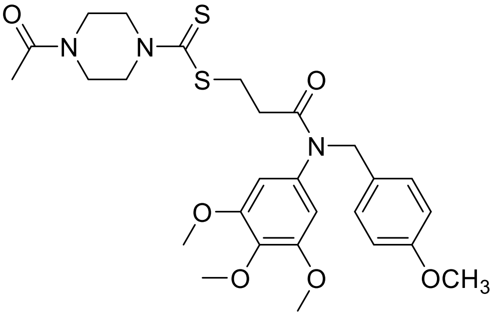

White powder, yield, 52.3 %, m.p:46~48 °C. ^{1}H NMR (400 MHz, $CDCl_3$) δ 7.08 (d, *J* = 8.6 Hz, 2H), 6.74 (d, *J* = 8.6 Hz, 2H), 6.04 (s, 2H), 4.70 (s, 2H), 3.92 (d, *J* = 25.2 Hz, 4H), 3.78 (s, 3H), 3.71 (s, 3H), 3.64 (d, *J* = 8.1 Hz, 8H), 3.59 – 3.43 (m, 4H), 2.52 (t, *J* = 7.0 Hz, 2H), 2.06 (s, 3H). ^{13}C NMR (100 MHz, $CDCl_3$) δ 169.82, 158.03, 152.52, 136.72, 136.18, 129.57, 128.75, 112.65, 104.87, 59.94, 55.18, 54.27, 51.39, 44.17, 39.54, 32.96, 31.53, 28.26, 20.34. HRMS calculated for $C_{27}H_{36}N_3O_6S_2$, $[M+H]^+$ m/z: 562.2046, found: 562.2052.

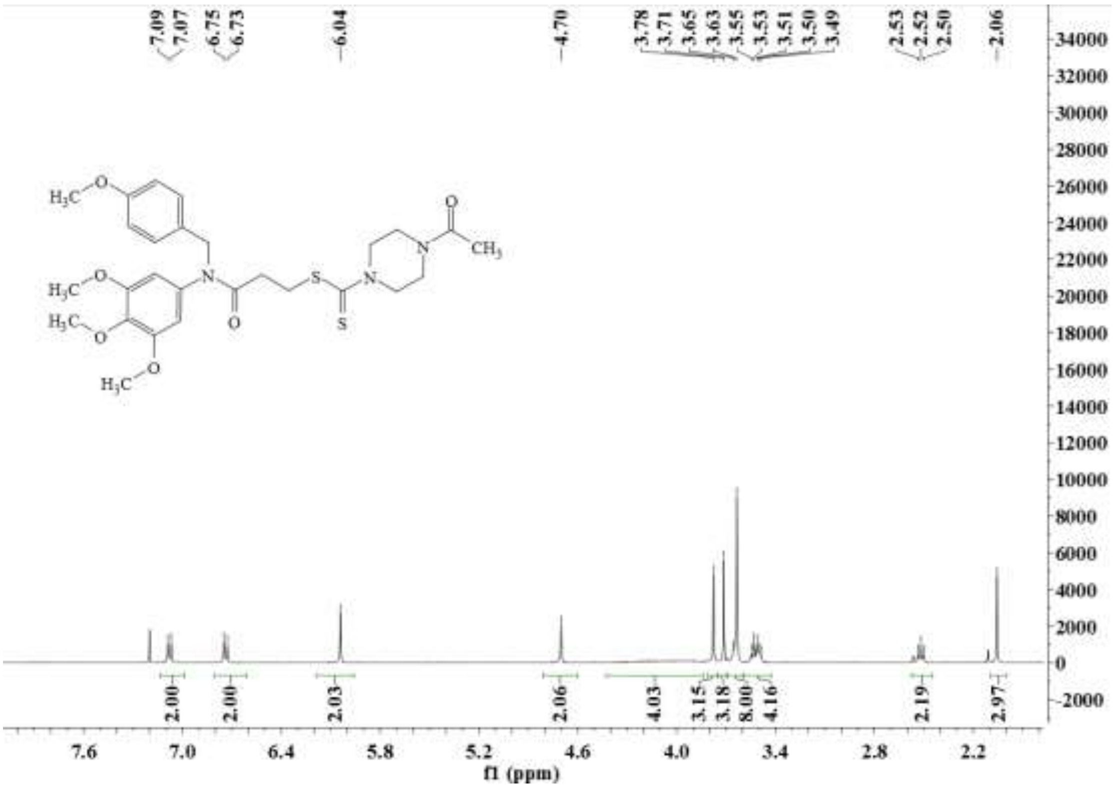

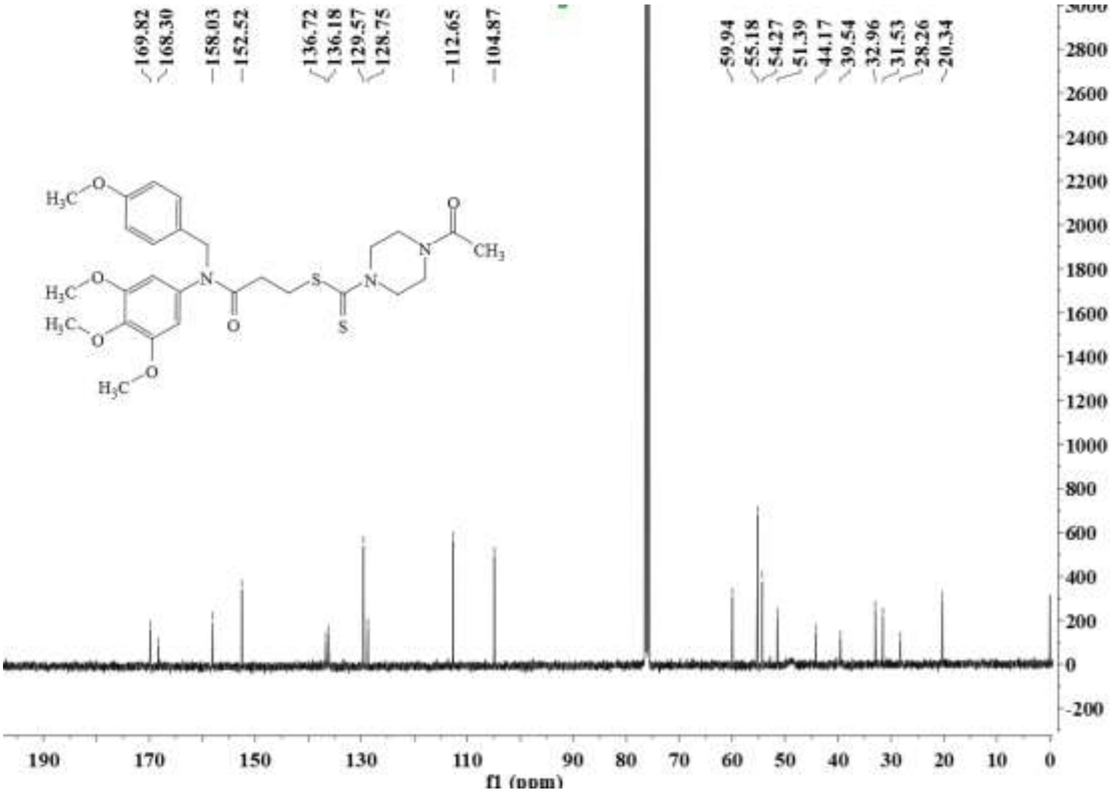

4-((4-methoxybenzyl)(3,4,5-trimethoxyphenyl)amino)-4-oxobutyl-4-methylpiperazine-1-carbodithioate (C5)

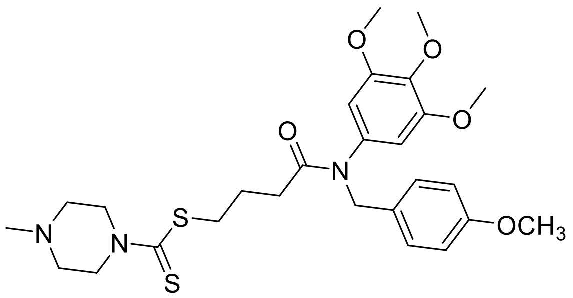

White powder, yield, 78.5 %, m.p:86~88 ºC. ^{1}H NMR (400 MHz, $CDCl_3$) δ 7.07 (d, *J* = 8.5 Hz, 2H), 6.73 (d, *J* = 8.5 Hz, 2H), 6.05 (s, 2H), 4.69 (s, 2H), 4.25 (s, 2H), 3.98 – 3.79 (m, 2H), 3.78 (s, 3H), 3.71 (s, 3H), 3.64 (s, 6H), 3.21 (t, *J* = 7.3 Hz, 2H), 2.39 (s, 4H), 2.25 (s, 3H), 2.17 (t, *J* = 7.2 Hz, 2H), 2.02 – 1.85 (m, 2H). ^{13}C NMR (100 MHz, $CDCl_3$) δ 195.90, 170.78, 157.96, 152.46, 136.67, 136.58, 129.51, 128.97, 112.64, 104.81, 59.93, 55.17, 54.26 53.34, 51.26, 44.59, 35.47, 32.28, 23.83. HRMS calculated for $C_{27}H_{38}N_3O_5S_2$, $[M+H]^+$ m/z: 548.2253, found: 548.2258.

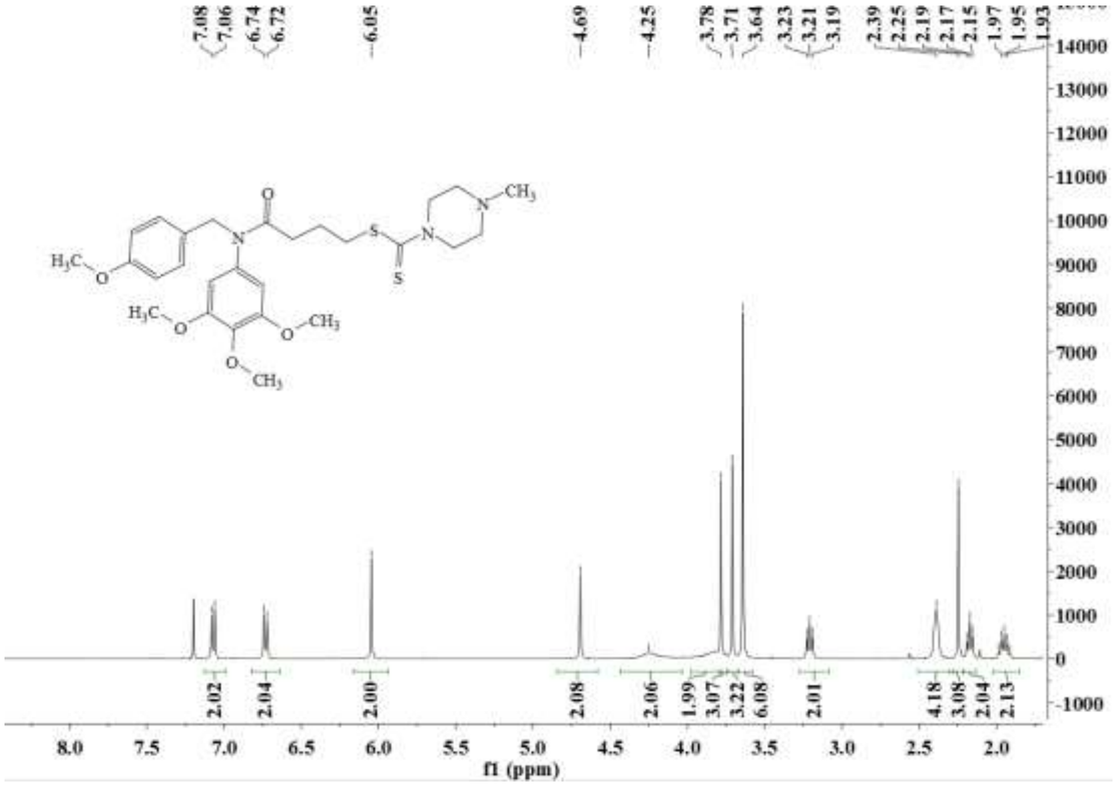

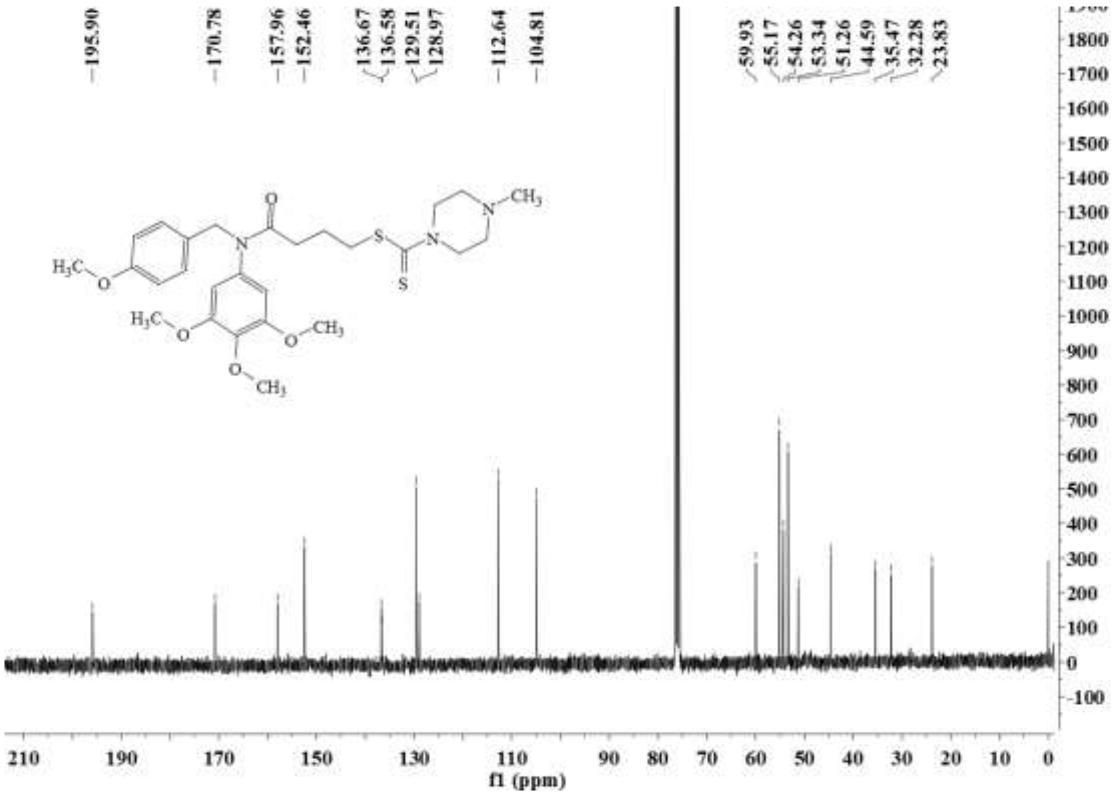

3-((4-methoxybenzyl)(3,4,5-trimethoxyphenyl)amino)-3-oxopropyl-4-methylpiperazine-1-carbodithioate (C6)

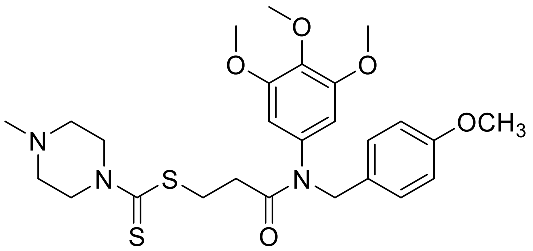

White powder, yield, 63.1 %, m.p:122~124 ^{o}C. ^{1}H NMR (400 MHz, $CDCl_3$) δ 7.08 (d, *J* = 8.6 Hz, 2H), 6.73 (d, *J* = 8.6 Hz, 2H), 6.04 (s, 2H), 4.70 (s, 2H), 4.23 (s, 2H), 3.88 (m, 2H), 3.77 (s, 3H), 3.71 (s, 3H), 3.63 (s, 6H), 3.52 (t, *J* = 7.0 Hz, 2H), 2.52 (t, *J* = 7.0 Hz, 2H), 2.46 – 2.30 (m, 4H), 2.24 (s, 3H). ^{13}C NMR (100 MHz, $CDCl_3$) δ 195.99, 169.97, 157.99, 152.49, 136.67, 136.24, 129.55, 128.83, 112.63, 104.87, 59.94, 55.16, 54.26, 53.36, 51.36, 44.60, 33.09, 31.48. HRMS calculated for $C_{26}H_{36}N_3O_5S_2$, $[M+H]^+$ m/z: 534.2096, found: 534.2102.

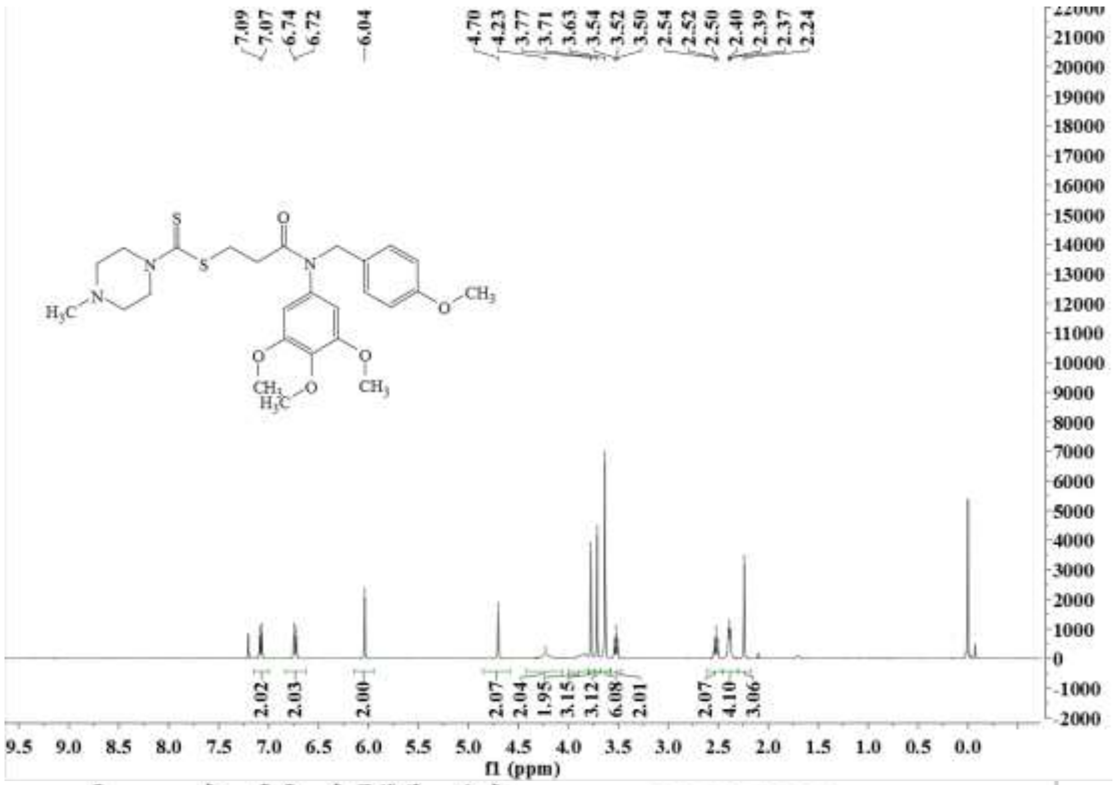

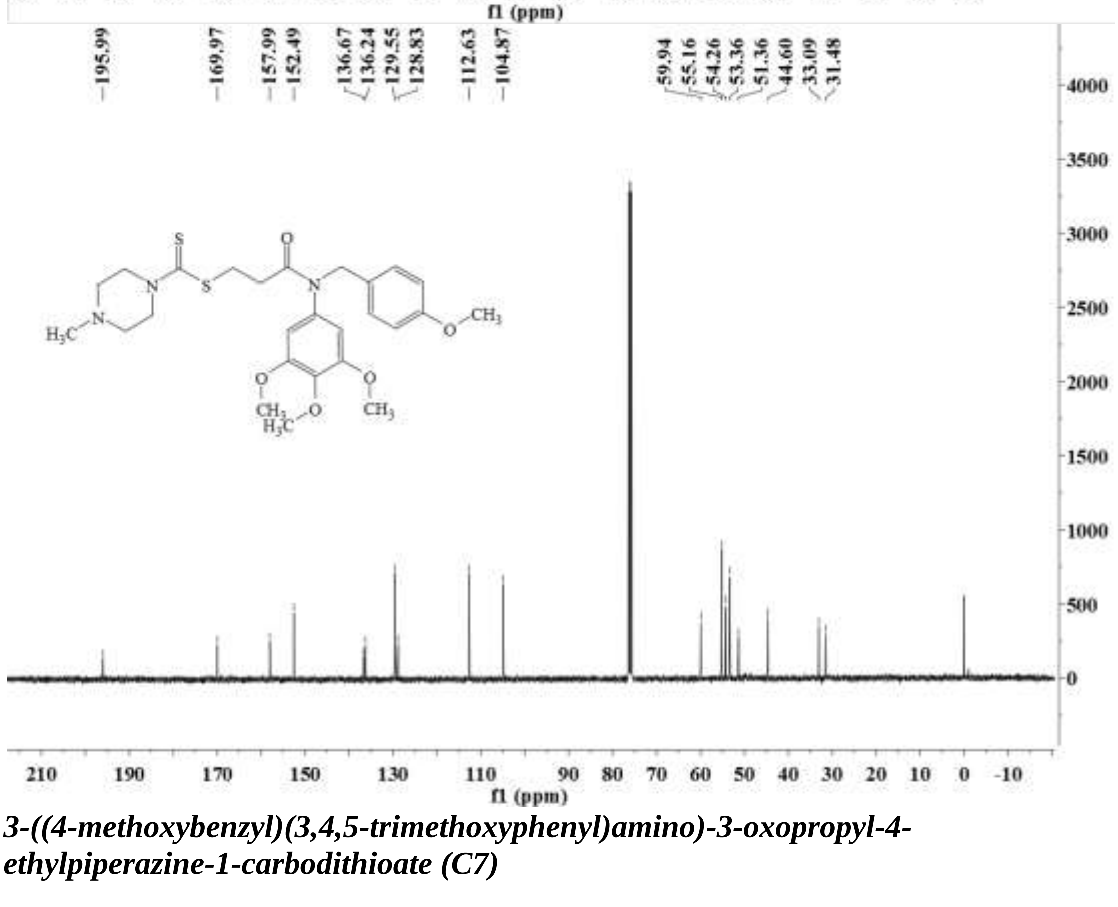

3-((4-methoxybenzyl)(3,4,5-trimethoxyphenyl)amino)-3-oxopropyl-4-ethylpiperazine-1-carbodithioate (C7)

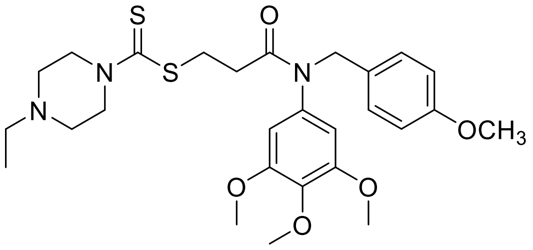

White powder, yield, 50.6 %, m.p:124~126 °C. ^{1}H NMR (400 MHz, $CDCl_3$) δ 7.08 (d, *J* = 8.6 Hz, 2H), 6.73 (d, *J* = 8.6 Hz, 2H), 6.04 (s, 2H), 4.70 (s, 2H), 4.23 (s, 2H), 3.88 (s, 2H), 3.77 (s, 3H), 3.71 (s, 3H), 3.63 (s, 6H), 3.52 (t, *J* = 7.0 Hz, 2H), 2.52 (t, *J* = 7.0 Hz, 2H), 2.42 (m, 4H), 2.37 (q, *J* = 7.2 Hz, 2H), 1.02 (t, *J* = 7.2 Hz, 3H). ^{13}C NMR (100 MHz, $CDCl_3$) δ 195.77, 169.98, 157.99, 152.48, 136.66, 136.24, 129.55, 128.84, 112.63, 104.87, 59.94, 55.16, 54.25, 51.36, 51.12, 50.88, 33.10, 31.47, 10.93. HRMS calculated for $C_{27}H_{38}N_3O_5S_2$, $[M+H]^+$ m/z: 548.2253, found: 548.2259.

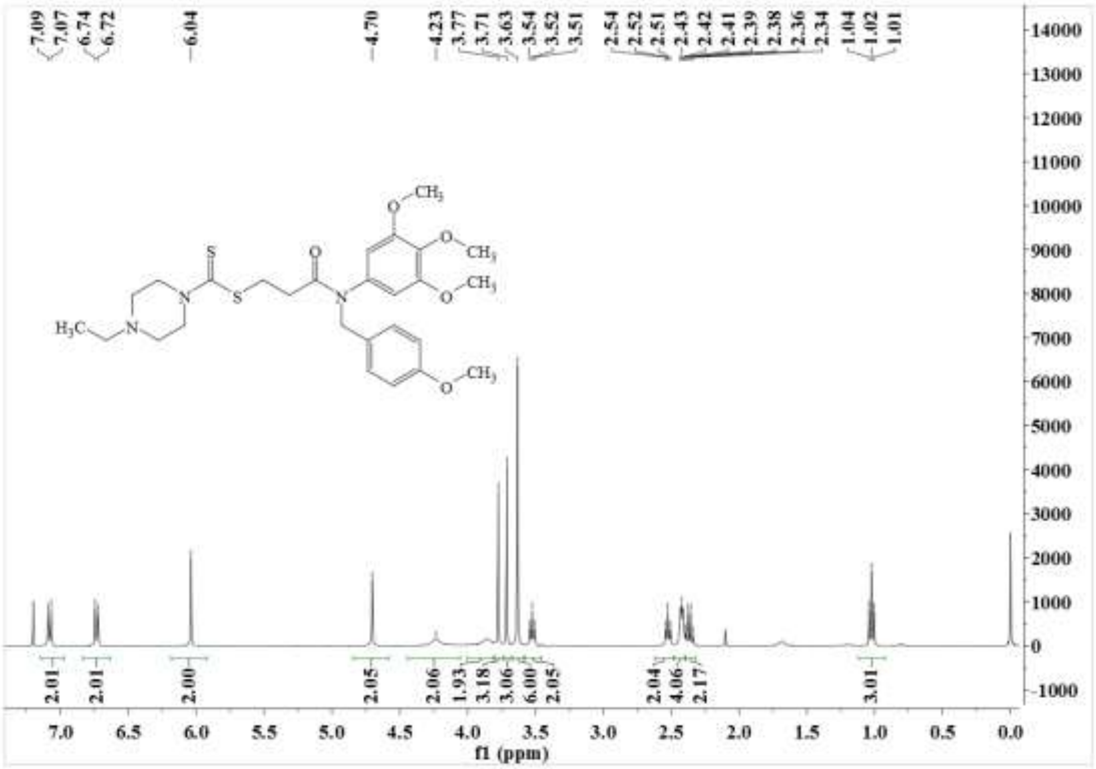

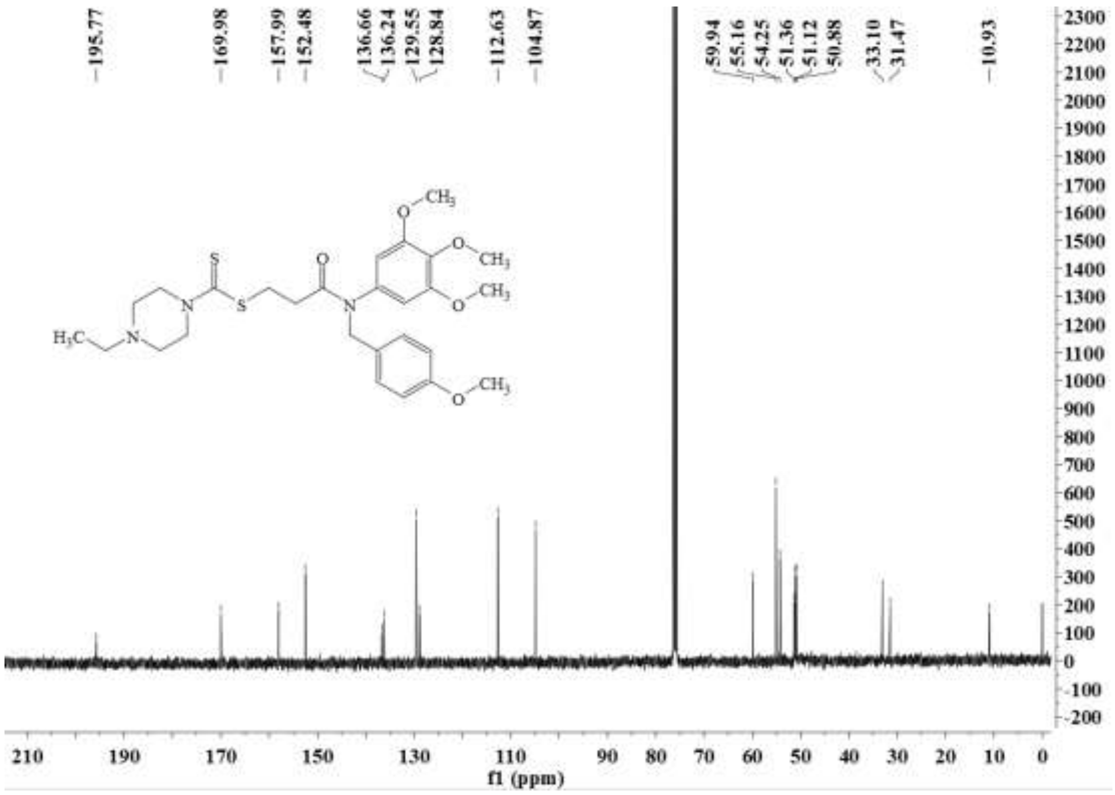

2-((4-methoxybenzyl)(3,4,5-trimethoxyphenyl)amino)-2-oxoethyl-4-ethylpiperazine-1-carbodithioate (C8)

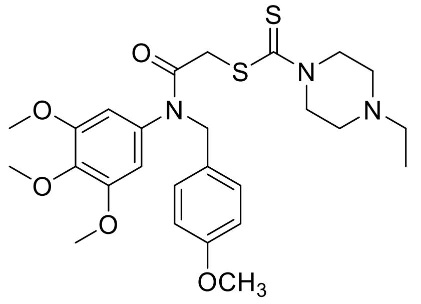

White powder, yield, 73.1 %, m.p:41~43 °C. ^{1}H NMR (400 MHz, $CDCl_3$) δ 7.10 (d, J = 8.6 Hz, 2H), 6.73 (d, J = 8.6 Hz, 2H), 6.26 (s, 2H), 4.74 (s, 2H), 4.27 (s, 2H), 3.94 (s, 4H), 3.78 (s, 3H), 3.71 (s, 3H), 3.66 (s, 6H), 2.53 (m, 4H), 2.44 (q, J = 7.2 Hz, 2H), 1.05 (t, J = 7.2 Hz, 3H). ^{13}C NMR (100 MHz, $CDCl_3$) δ 195.03, 165.69, 158.04, 152.51, 136.90, 135.85, 129.60, 128.52, 112.64, 104.96, 59.94, 55.19, 54.26, 52.05, 50.83, 40.03, 28.26, 10.61. HRMS calculated for $C_{26}H_{36}N_3O_5S_2$, $[M+H]^+$ m/z: 534.2096, found: 534.2104.

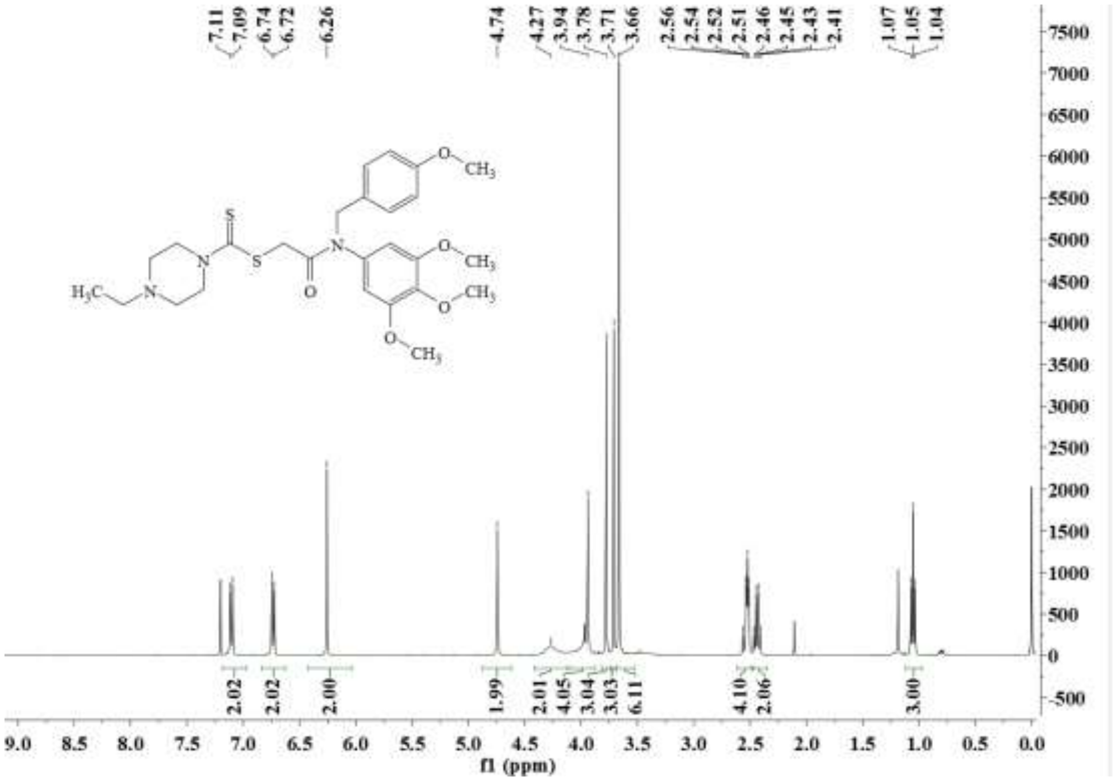

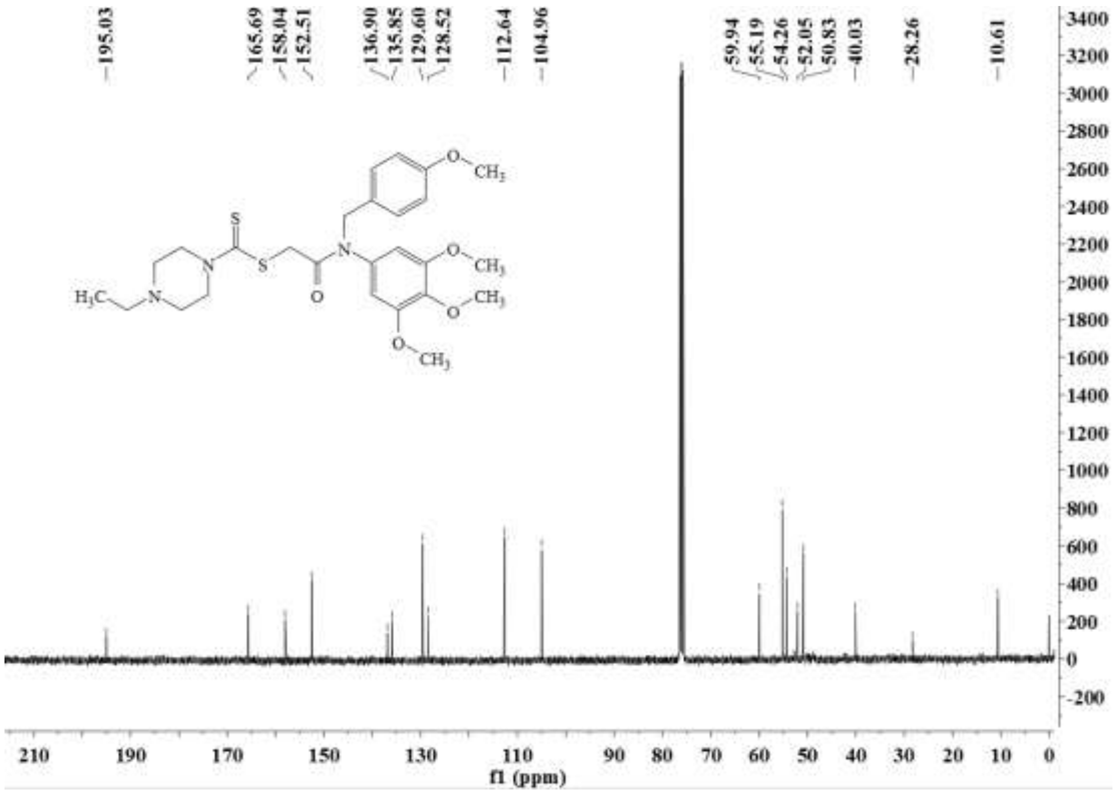

4-((4-methoxybenzyl)(3,4,5-trimethoxyphenyl)amino)-4-oxobutyl-4-ethylpiperazine-1-carbodithioate (C9)

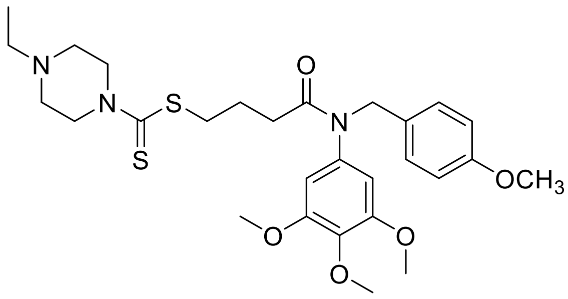

Yellow liquid, yield, 52.9 %. ^{1}H NMR (400 MHz, $CDCl_3$) δ 7.07 (d, *J* = 8.6 Hz, 2H), 6.73 (d, *J* = 8.6 Hz, 2H), 6.05 (s, 2H), 4.69 (s, 2H), 4.26 (s, 2H), 3.85 (s, 2H), 3.78 (s, 3H), 3.71 (s, 3H), 3.64 (s, 6H), 3.21 (t, *J* = 7.4 Hz, 2H), 2.42 (s, 4H), 2.38 (m, 2H), 2.17 (t, *J* = 7.2 Hz, 2H), 1.99 (m, 2H), 1.03 (t, *J* = 7.2 Hz, 3H). ^{13}C NMR (100 MHz, $CDCl_3$) δ 195.71, 170.78, 157.96, 152.47, 136.67, 129.51, 128.97, 112.65, 104.81, 59.94, 55.17, 54.26, 51.26, 51.08, 50.87, 35.45, 32.28, 23.84, 10.88. HRMS calculated for $C_{28}H_{40}N_3O_5S_2$, $[M+H]^+$ m/z: 562.2409, found: 562.2413.

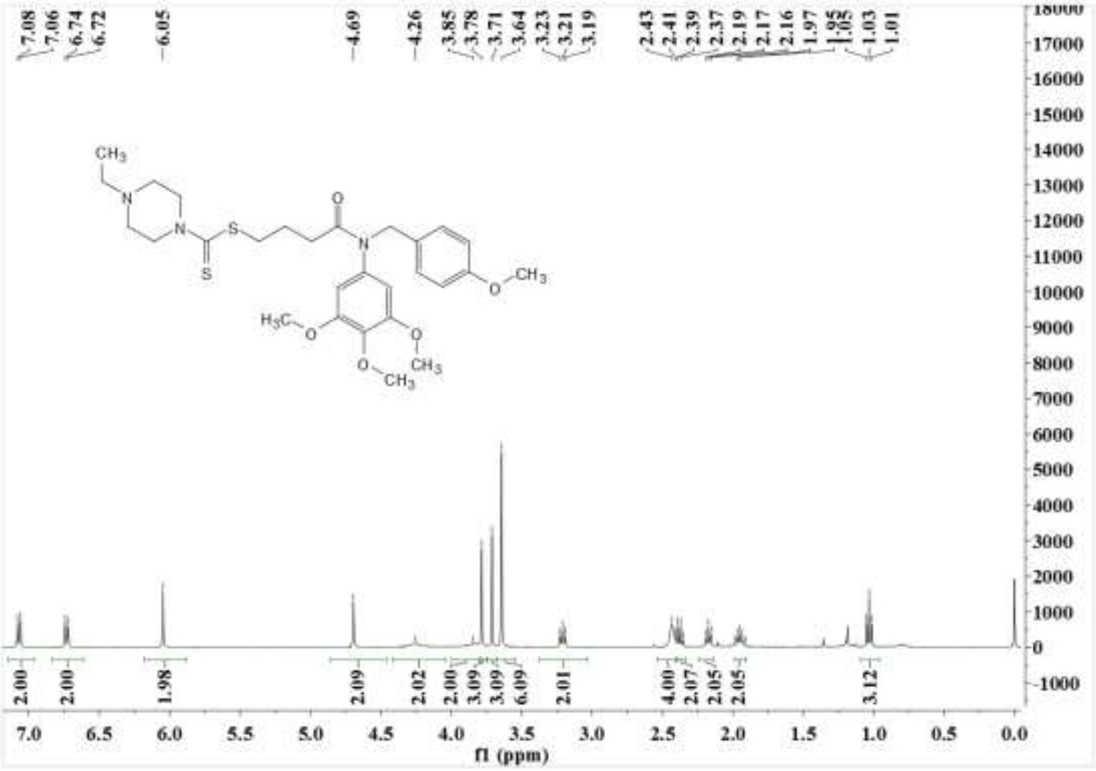

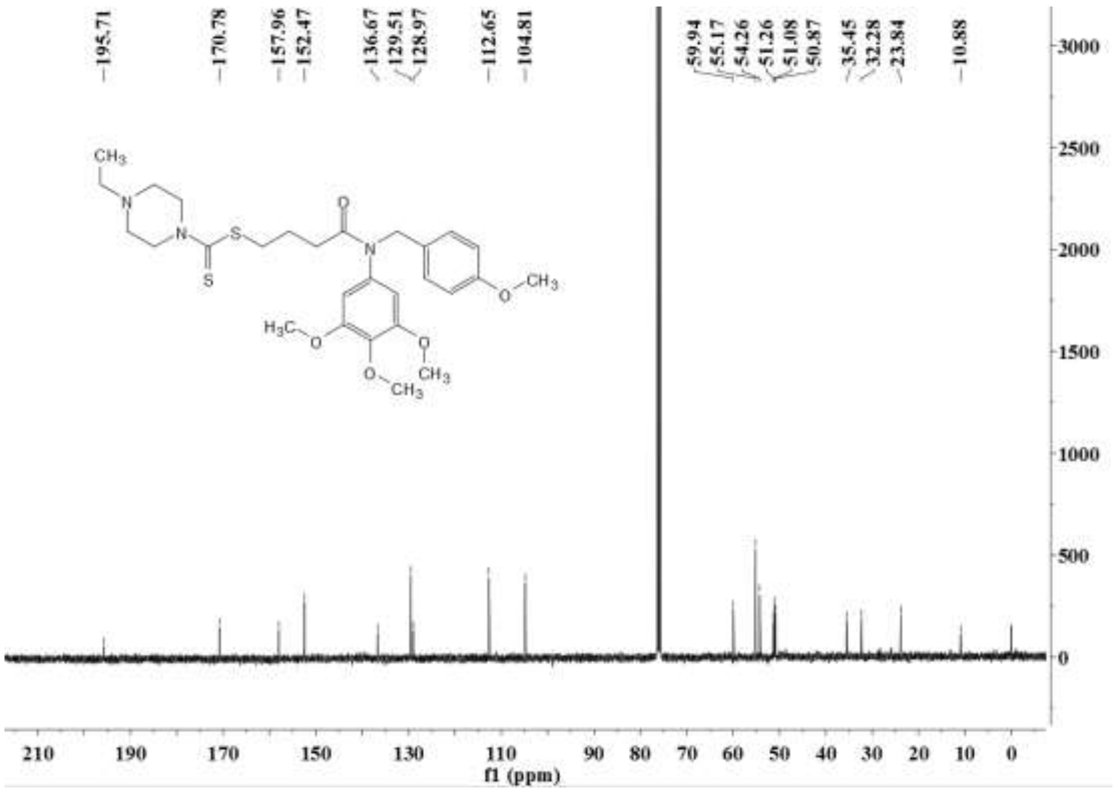

2-((4-methoxybenzyl)(3,4,5-trimethoxyphenyl)amino)-2-oxoethyl-pyrrolidine-1-carbodithioate (C10)

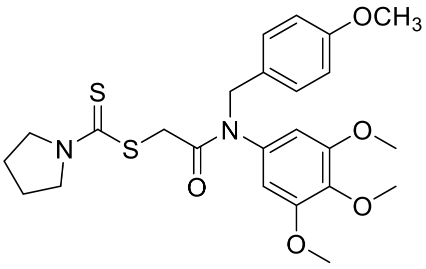

White powder, yield, 80.2 %, m.p:150~152 °C. ^{1}H NMR (400 MHz, $CDCl_3$) δ 7.11 (d, *J* = 8.5 Hz, 2H), 6.73 (d, *J* = 8.5 Hz, 2H), 6.27 (s, 2H), 4.75 (s, 2H), 3.94 (s, 2H), 3.83 (t, *J* = 7.0 Hz, 2H), 3.78 (s, 3H), 3.71 (s, 3H), 3.67 (s, 6H), 3.65 (d, *J* = 7.1 Hz, 2H), 2.07 – 1.95 (m, 2H), 1.90 (p, *J* = 6.7 Hz, 2H). ^{13}C NMR (100 MHz, $CDCl_3$) δ 190.78, 165.92, 158.02, 152.49, 136.86, 135.91, 129.59, 128.58, 112.64, 105.01, 59.93, 55.20, 54.25, 54.23, 52.07, 49.74, 39.52, 25.13, 23.32. HRMS calculated for $C_{24}H_{31}N_2O_5S_2$, $[M+H]^+$ m/z: 491.1674, found: 491.1678.

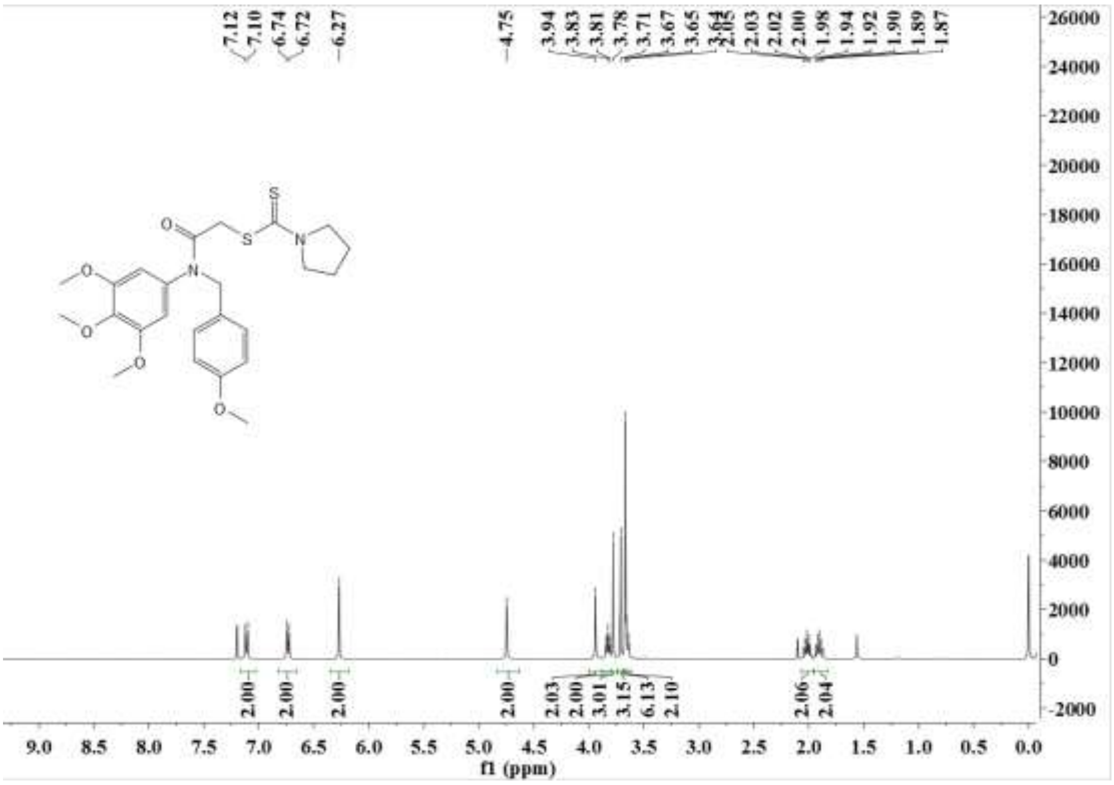

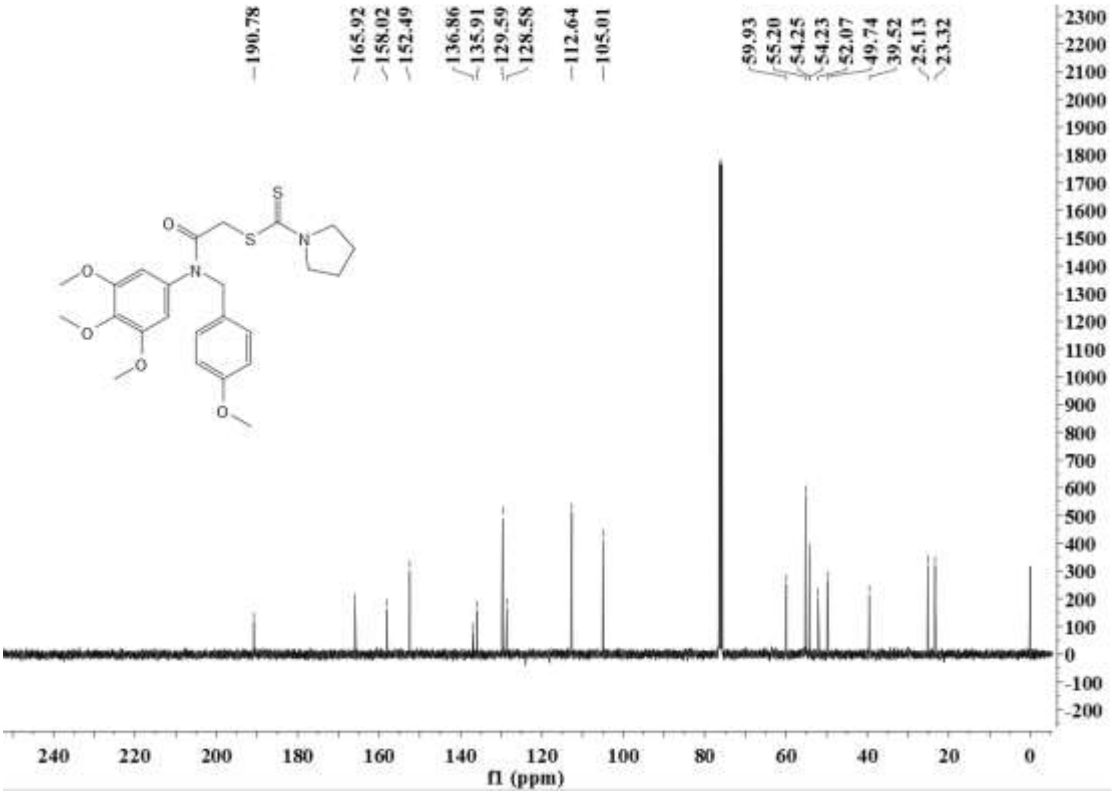

2-((4-methoxybenzyl)(3,4,5-trimethoxyphenyl)amino)-2-oxoethylpiperidine-1-carbodithioate (C11)

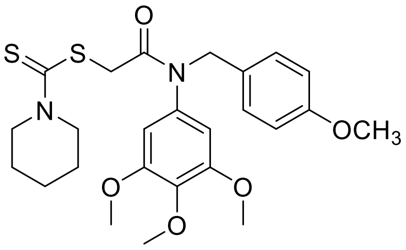

White powder, yield, 63.4 %, m.p:138~140 °C. ^{1}H NMR (400 MHz, $CDCl_3$) δ 7.11 (d, *J* = 8.5 Hz, 2H), 6.73 (d, *J* = 8.6 Hz, 2H), 6.27 (s, 2H), 4.75 (s, 2H), 4.18 (s, 2H), 3.94 (s, 2H), 3.83 (s, 2H), 3.77 (s, 3H), 3.71 (s, 3H), 3.66 (s, 6H), 1.63 (s, 6H). ^{13}C NMR (100 MHz, $CDCl_3$) δ 193.65, 165.92, 158.01, 152.48, 136.84, 135.94, 129.59, 128.62, 112.63, 104.99, 59.93, 55.19, 54.25, 52.01, 40.06, 23.19. HRMS calculated for $C_{25}H_{33}N_2O_5S_2$, $[M+H]^+$ m/z: 505.1831, found: 505.1839.

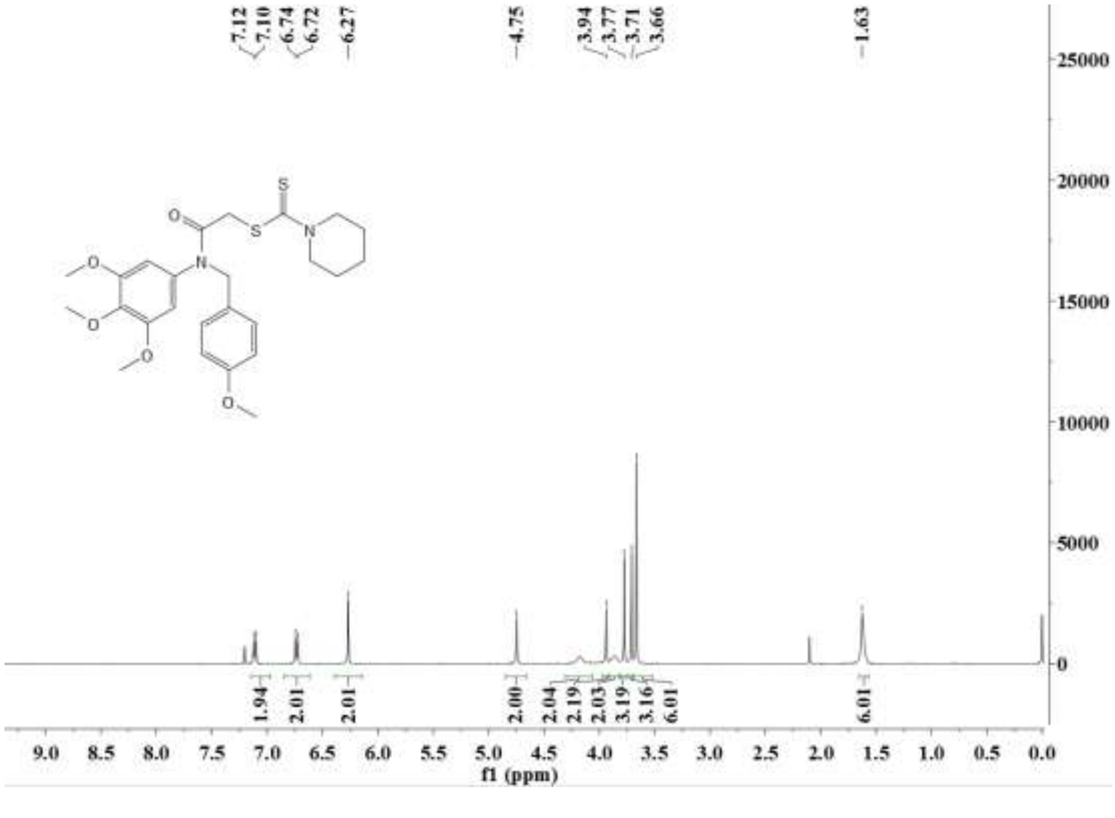

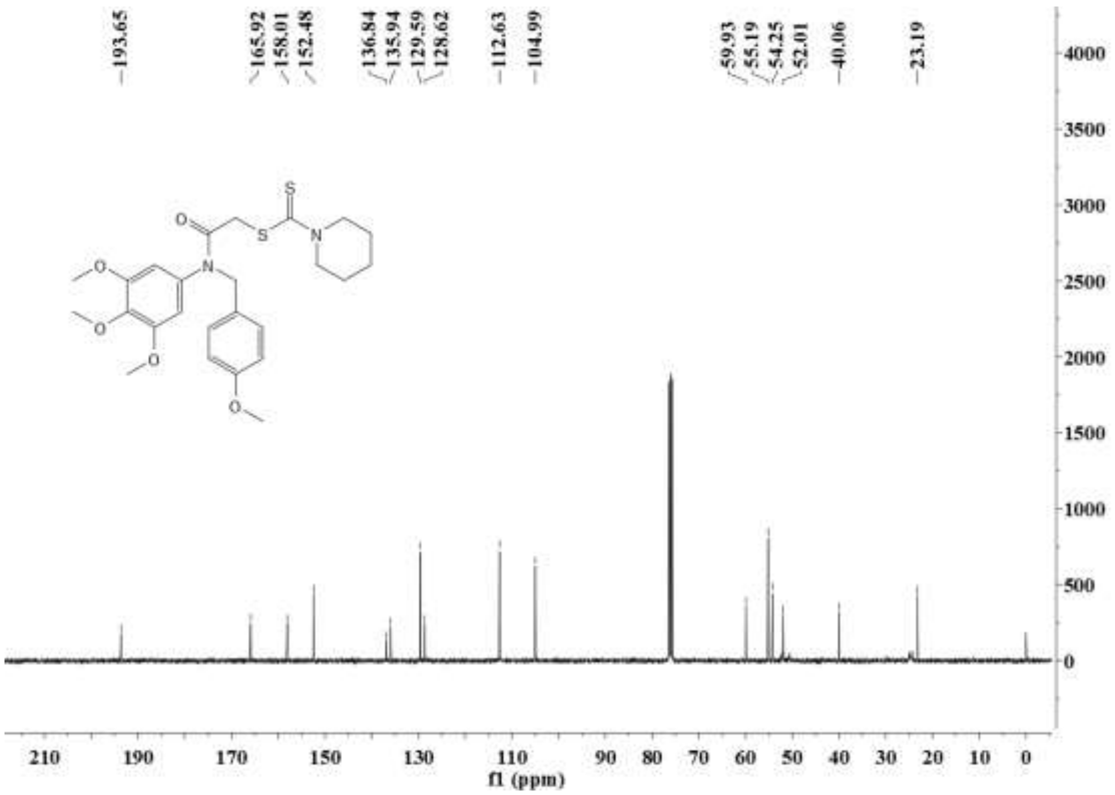

2-((4-methoxybenzyl)(3,4,5-trimethoxyphenyl)amino)-2-oxoethyl-3-methylpiperidine-1-carbodithioate (C12)

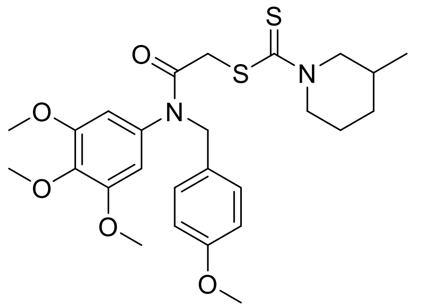

White powder, yield, 48.6 %, m.p:113~115 °C. ^{1}H NMR (400 MHz, $CDCl_3$) δ 7.11 (d, *J* = 8.5 Hz, 2H), 6.73 (d, *J* = 8.5 Hz, 2H), 6.27 (s, 2H), 5.22 (d, *J* = 10.7 Hz, 1H), 4.75 (d, *J* = 9.0 Hz, 2H), 4.45 (dd, 1H), 4.12 (m, 2H), 3.78 (s, 3H), 3.71 (s, 3H), 3.66 (s, 6H), 3.09 (s, 1H), 2.77 (m, 1H), 1.79 (d, *J* = 13.1 Hz, 1H), 1.68 (s, 2H), 1.60 (m, 1H), 1.18 (m, 1H), 0.88 (d, *J* = 6.5 Hz, 3H). ^{13}C NMR (100 MHz, $CDCl_3$) δ 193.67, 165.91, 158.01, 152.47, 136.83, 135.93, 129.59, 128.61, 112.62, 104.97, 59.93, 55.18, 54.25, 52.01, 40.10, 31.79, 17.74. HRMS calculated for $C_{26}H_{35}N_2O_5S_2$, $[M+H]^+$ m/z: 519.1987, found: 519.1996.

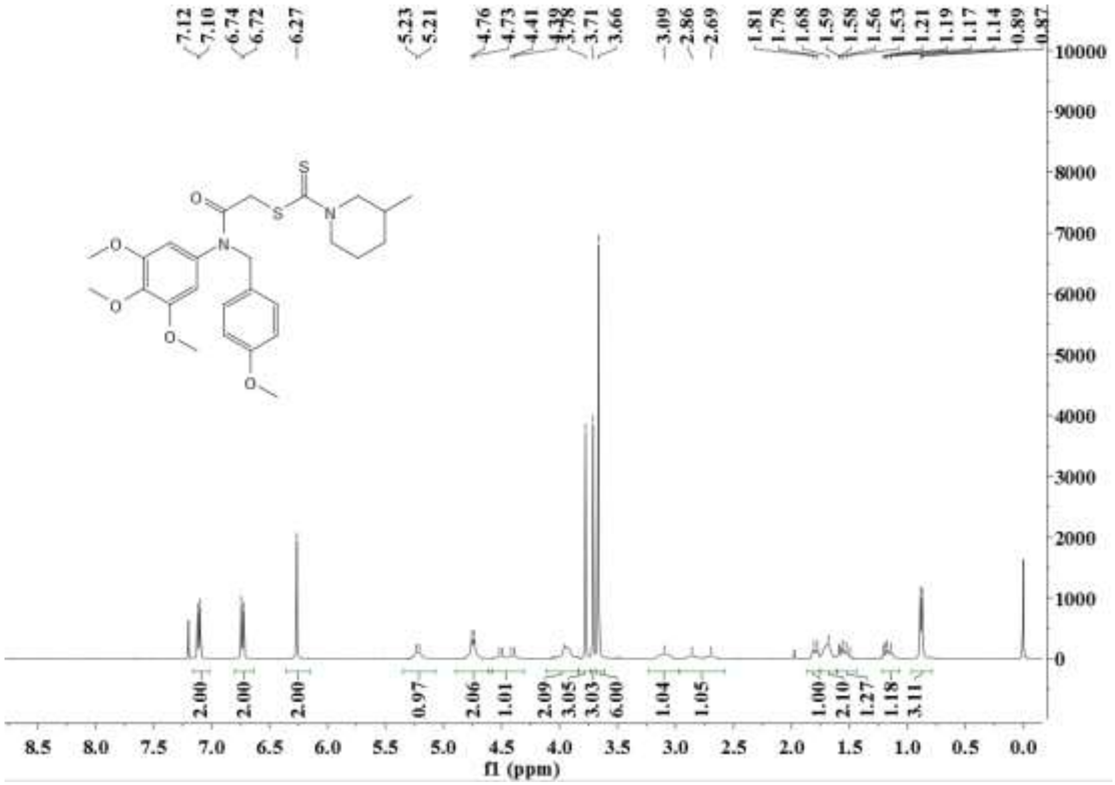

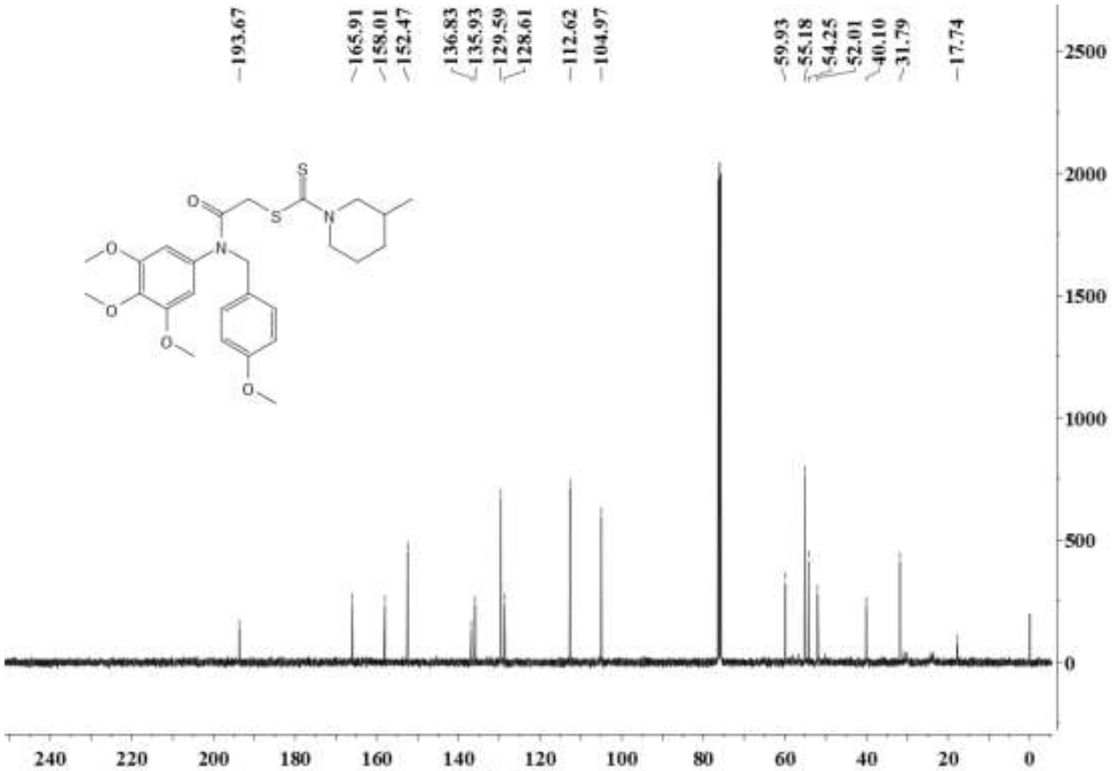

2-((4-methoxybenzyl)(3,4,5-trimethoxyphenyl)amino)-2-oxoethyl-morpholine-4-carbodithioate (C13)

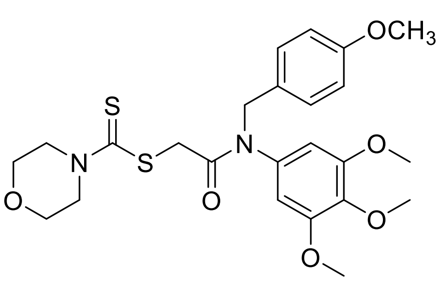

White powder, yield, 70.1 %, m.p:163~165 °C. ^{1}H NMR (400 MHz, $CDCl_3$) δ 7.11 (d, *J* = 8.5 Hz, 2H), 6.74 (d, *J* = 8.6 Hz, 2H), 6.26 (s, 2H), 4.74 (s, 2H), 4.21 (s, 2H), 3.95 (s, 4H), 3.78 (s, 3H), 3.71 (s, 3H), 3.69 (m, 4H), 3.67 (s, 6H). ^{13}C NMR (100 MHz, $CDCl_3$) δ 195.68, 165.57, 158.05, 152.53, 136.91, 135.82, 129.61, 128.49, 112.64, 104.94, 65.17, 59.94, 55.19, 54.25, 52.07, 39.86. HRMS calculated for $C_{24}H_{31}N_2O_6S_2$, $[M+H]^+$ m/z: 507.1624, found: 507.1629.

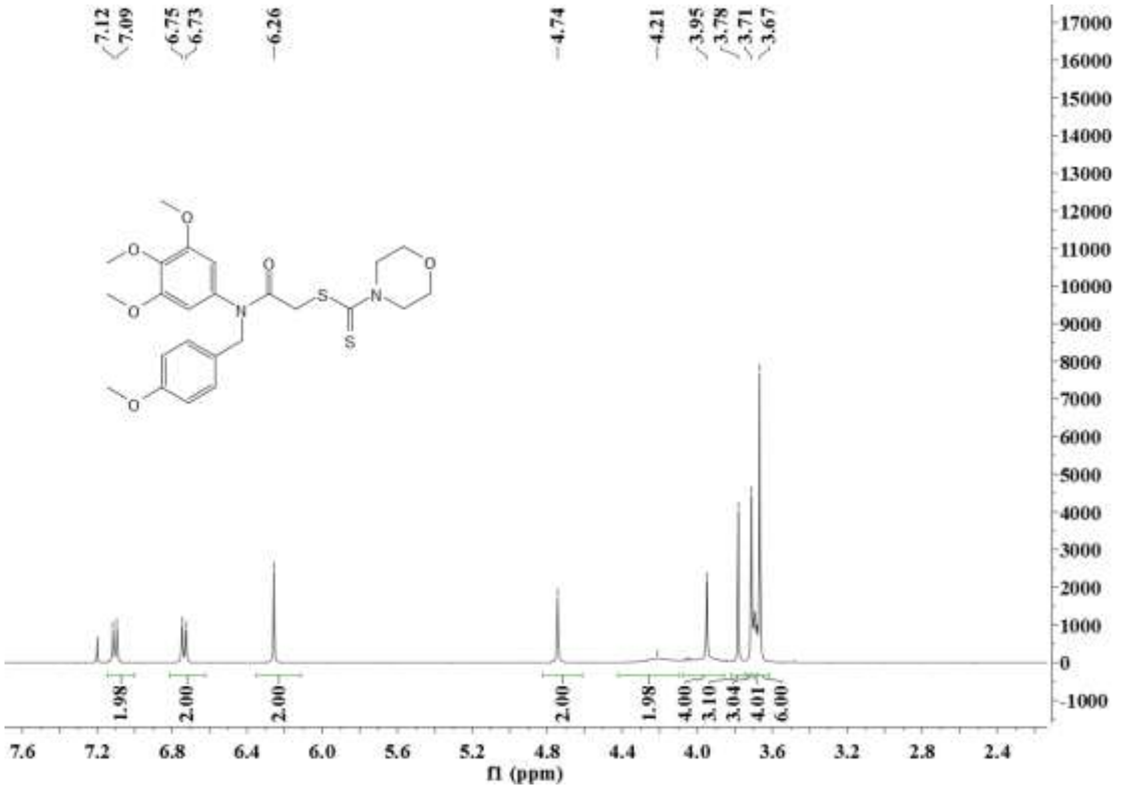

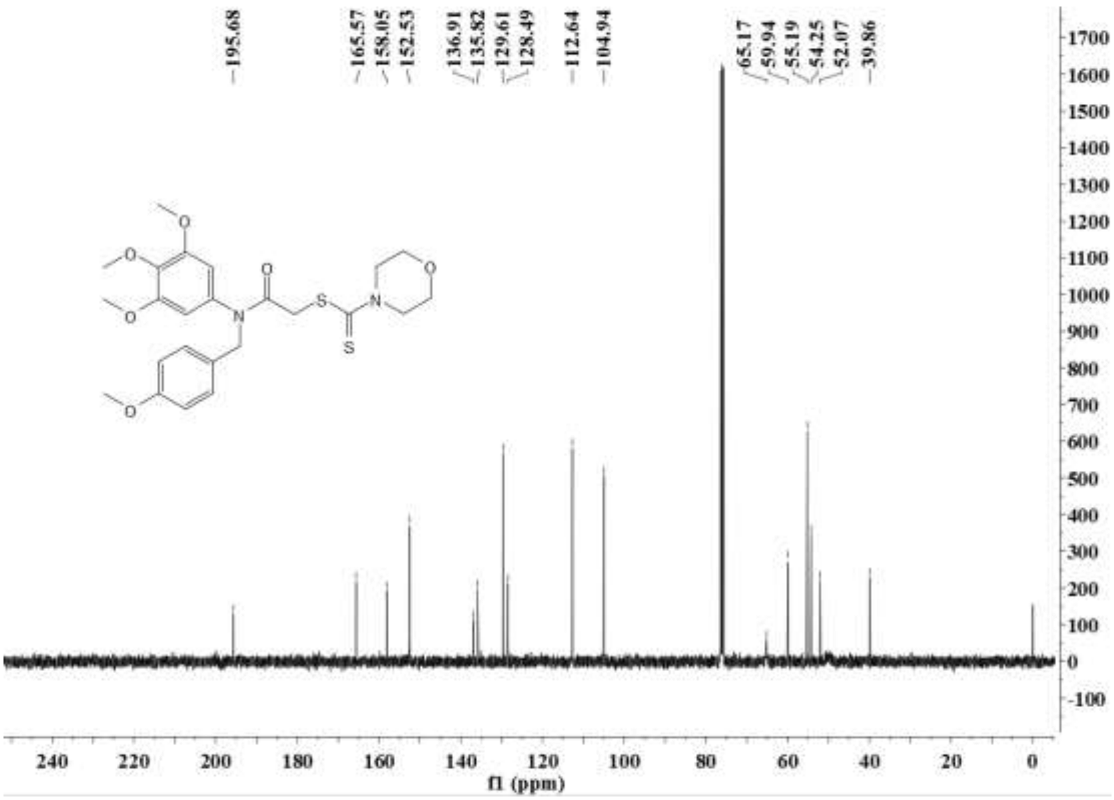

2-((4-methoxybenzyl)(3,4,5-trimethoxyphenyl)amino)-2-oxoethyl-3,5-dimethylpiperidine-1-carbodithioate (C14)

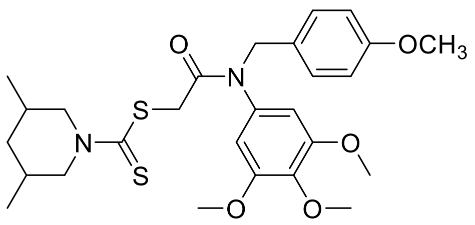

White powder, yield, 51.3 %, m.p:128~130 ^oC. ^1H NMR (400 MHz, $CDCl_3$) δ 7.11 (d, *J* = 8.5 Hz, 2H), 6.73 (d, *J* = 8.5 Hz, 2H), 6.26 (s, 2H), 5.41 (d, *J* = 10.7 Hz, 1H), 4.87 – 4.60 (m, 2H), 4.52 (d, *J* = 12.4 Hz, 1H), 4.11 – 3.83 (m, 2H), 3.78 (s, 3H), 3.71 (s, 3H), 3.66 (s, 6H), 2.61 (t, *J* = 12.0 Hz, 1H), 2.40 (t, *J* = 11.8 Hz, 1H), 1.79 (d, *J* = 13.0 Hz, 1H), 1.74 – 1.58 (m, 2H), 0.88 (s, 3H), 0.86 (s, 3H), 0.79 (m, 1H). ^{13}C NMR (100 MHz, $CDCl_3$) δ 193.48, 165.91, 158.01, 152.47, 136.83, 135.92, 129.59, 128.59, 112.62, 104.96, 59.93, 57.89, 56.34, 55.18, 54.25, 52.01, 41.12, 40.14, 30.74, 29.86, 17.81. HRMS calculated for $C_{27}H_{37}N_2O_5S_2$, $[M+H]^+$ m/z: 533.2144, found: 533.2148.

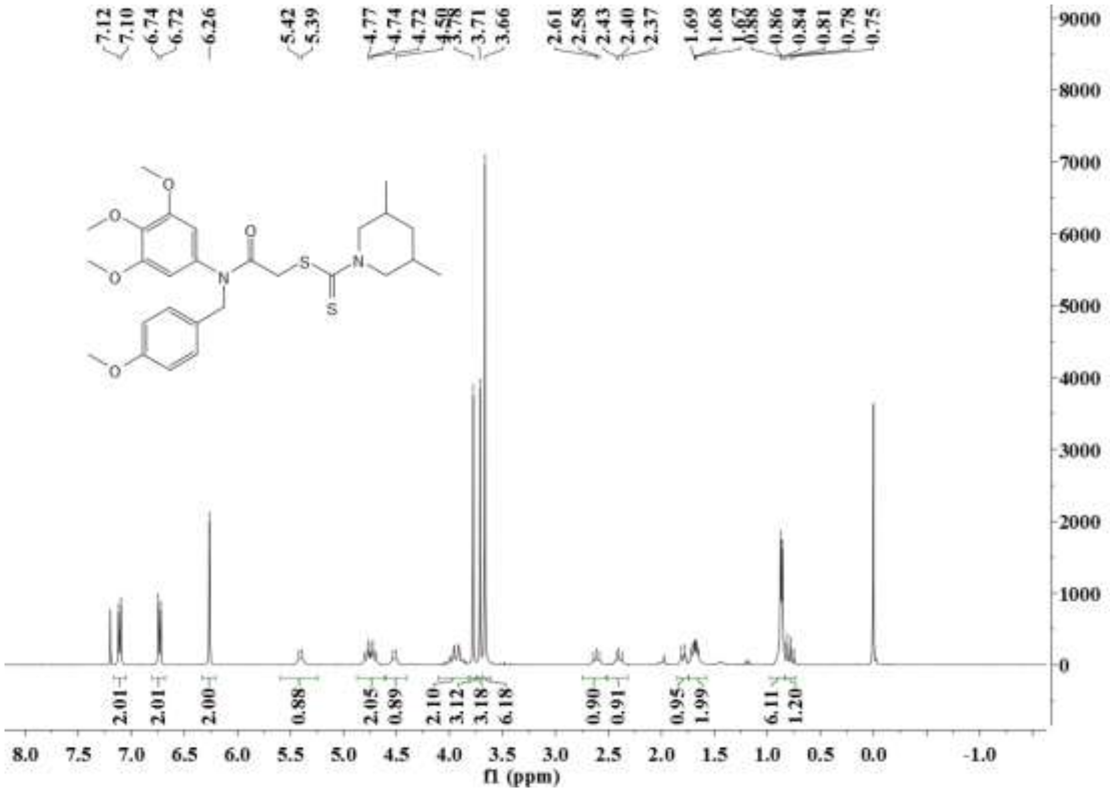

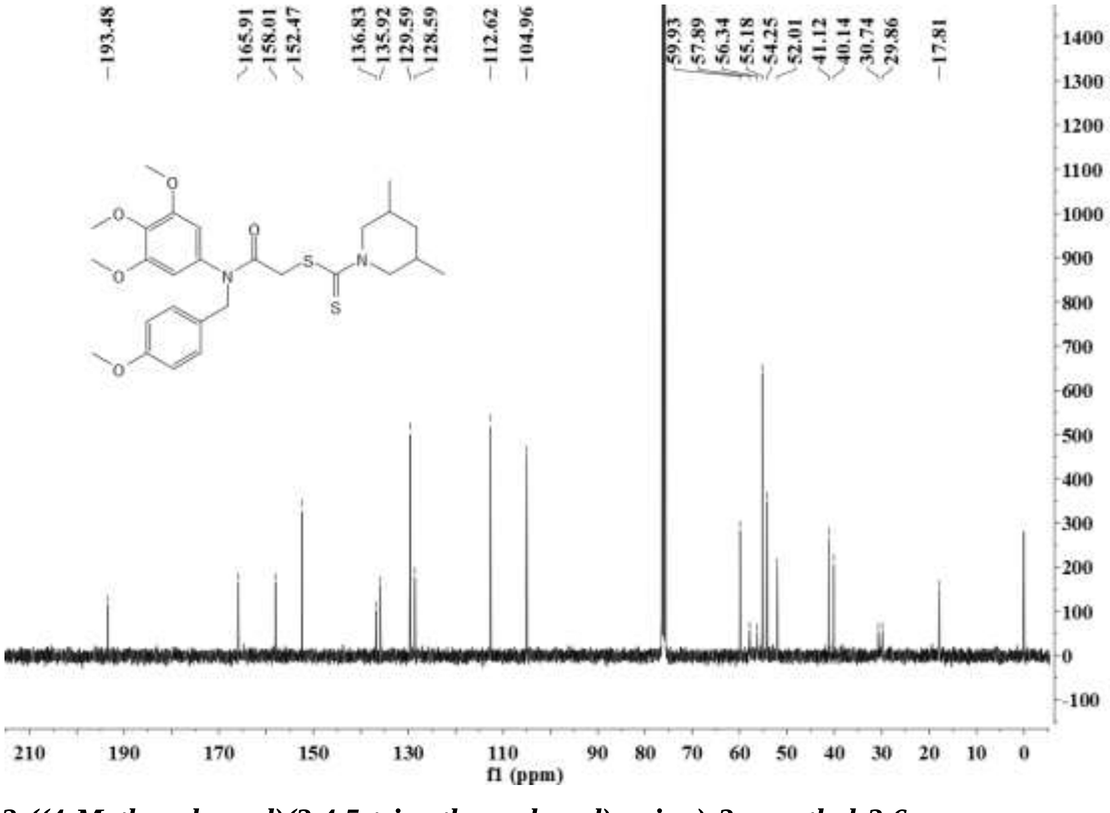

2-((4-Methoxybenzyl)(3,4,5-trimethoxyphenyl)amino)-2-oxoethyl-2,6-dimethylpiperidine-1-carbodithioate (C15)

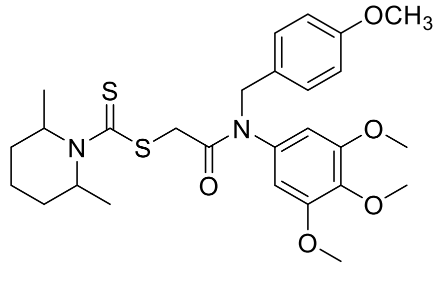

White powder, yield, 76.2 %, m.p:124~126 °C. ^{1}H NMR (400 MHz, $CDCl_3$) δ 7.12 (d, *J* = 8.5 Hz, 2H), 6.73 (d, *J* = 8.5 Hz, 2H), 6.27 (s, 2H), 5.75 – 5.50 (m, 1H), 5.00 – 4.82 (m, 1H), 4.82 – 4.66 (m, 2H), 4.10 (s, 1H), 3.81 (s, 1H), 3.77 (s, 3H), 3.71 (s, 3H), 3.67 (s, 6H), 1.83 – 1.48 (m, 6H), 1.31 (d, *J* = 7.0 Hz, 3H), 1.23 (d, *J* = 7.1 Hz, 3H). ^{13}C NMR (100 MHz, $CDCl_3$) δ 194.73, 166.04, 158.00, 152.47, 136.81, 136.01, 129.58, 128.66, 112.62, 104.97, 59.93, 55.18, 54.25, 53.12, 52.25, 51.98, 39.85, 29.32, 29.11, 18.87, 17.59, 12.81. HRMS calculated for $C_{27}H_{37}N_2O_5S_2$, $[M+H]^+$ m/z: 533.2144, found: 533.2150.

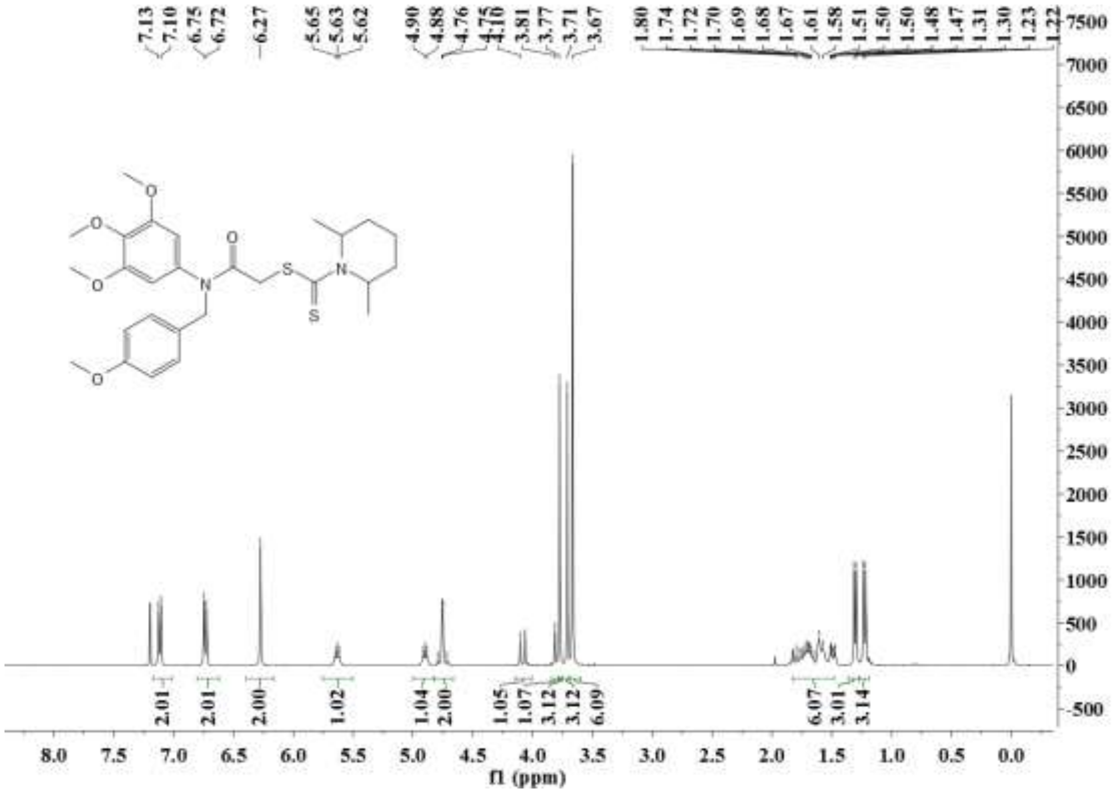

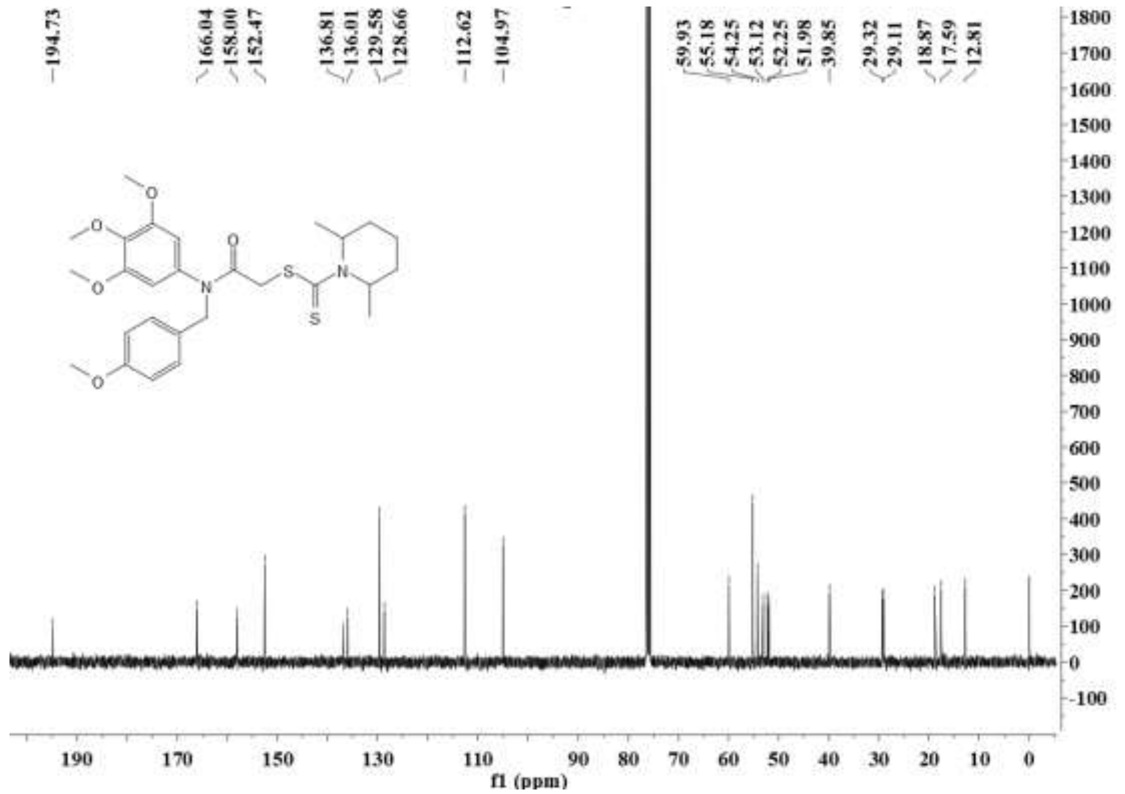

194.73
166.04
158.00
152.47
136.81
136.01
129.58
128.66
112.62
104.97
59.93
55.18
54.25
53.12
52.25
51.98
39.85
29.32
29.11
18.87
17.59
12.81
f1 (ppm)
